# Semisynthesis and Inhibitory Effects of Solidagenone Derivatives on TLR-Mediated Inflammatory Responses

**DOI:** 10.3390/molecules23123197

**Published:** 2018-12-04

**Authors:** Irene Cuadrado, Ángel Amesty, Juan Carlos Cedrón, Juan Carlos Oberti, Ana Estévez-Braun, Sonsoles Hortelano, Beatriz de las Heras

**Affiliations:** 1 Departamento de Farmacología, Farmacognosia y Botánica. Facultad de Farmacia, Universidad Complutense de Madrid (UCM), Plaza Ramón y Cajal s/n. 28040 Madrid, Spain; icberrocal@ucm.es; 2 Departamento de Química Orgánica, Instituto Universitario de Bio-Orgánica Antonio González, Universidad de La Laguna. Avda. Astrofísico Fco. Sánchez 2. La Laguna, 38206 Tenerife, Spain; angelamesty@yahoo.es (A.A.); jccedron@ull.es (J.C.C.); jco@fcq.unc.edu.ar (J.C.O.); 3 Facultad de Ciencias Químicas, Universidad de Córdoba and IMBIV (UNC−CONICET), Avenida Haya de la Torre y M. Allende, Ciudad Universitaria, X5000HUA Córdoba, Argentina; 4 Unidad de Terapias Farmacológicas. Área de Genética Humana. Instituto de Investigación de Enfermedades Raras (IIER), Instituto de Salud Carlos III, Carretera de Majadahonda-Pozuelo Km 2, 28220 Madrid, Spain

**Keywords:** solidagenone derivatives, diterpenes, inflammation, TLR4, molecular docking

## Abstract

A series of nine derivatives (**2**–**10**) were prepared from the diterpene solidagenone (**1**) and their structures were elucidated by means of spectroscopic studies. Their ability to inhibit inflammatory responses elicited in peritoneal macrophages by TLR ligands was investigated. Compounds **5** and **6** showed significant anti-inflammatory effects, as they inhibited the protein expression of nitric oxide synthase (NOS-2), cyclooxygenase-2 (COX-2), and cytokine production (TNF-α, IL-6, and IL-12) induced by the ligand of TLR4, lipopolysaccharide (LPS), acting at the transcriptional level. Some structure–activity relationships were outlined. Compound **5** was selected as a representative compound and molecular mechanisms involved in its biological activity were investigated. Inhibition of NF-κB and p38 signaling seems to be involved in the mechanism of action of compound **5**. In addition, this compound also inhibited inflammatory responses mediated by ligands of TLR2 and TLR3 receptors. To rationalize the obtained results, molecular docking and molecular dynamic studies were carried out on TLR4. All these data indicate that solidagenone derivative **5** might be used for the design of new anti-inflammatory agents.

## 1. Introduction

Inflammation is a protective physiological response of body tissues to harmful stimuli such as microbial infection or tissue injury. Nevertheless, an imbalance in the mechanisms that govern this process lead to the development of several pathologies [1,2]. The triggers and mechanisms leading to inflammation may vary between clinical conditions, but they share many common mediators, including specific patterns of eicosanoid and cytokine production.

The inflammatory response is initiated by the cellular sensing of either pathogen-associated molecular patterns (PAMPs) or damage-associated molecular patterns (DAMPs) through pattern recognition receptors (PRRs), such as Toll-like receptors (TLR), which trigger specific signaling pathways. During the inflammatory process, macrophages, being critical role players, dominate in the context of tissue injury and in the subsequent tissue repair [3,4]. Upon sensing the presence of pathogens through TLRs and other receptors, macrophages are stimulated to secrete a battery of cytokines that recruit effector cells into the infected area [5,6]. Most TLRs use the myeloid differentiation primary-response protein 88 (MyD88), a TIR (Toll/IL-1 receptor)-containing adaptor, to trigger a signaling pathway that culminates in the activation of downstream signaling pathways, such as the nuclear transcription factor-κB (NF-κB) pathway [7,8]. Activation of NF-κB leads to the upregulation of proinflammatory enzymes such as nitric oxide synthase-2 (NOS-2) and cyclooxygenase-2 (COX-2) and the production of inflammatory mediators such as nitric oxide (NO), prostaglandins, chemokines, and inflammatory cytokines that are well-known to be involved in the pathogenesis of inflammatory response. These signals are essential for the classical outcome of TLR activation: the orchestration of the host’s innate and adaptive immune responses. However, a persistent activation of the TLR-mediated intracellular signal transduction pathway in monocytes/macrophages, characterized by the excessive release of proinflammatory cytokines, maintains a chronic inflammatory state and may lead to the development of numerous pathologies [2,6]. Thus, control of macrophage activation and suppression and/or inhibition of the abovementioned signaling molecules may have great potential for preventing and treating inflammation-associated diseases.

Traditionally, natural products have represented an excellent resource for drug discovery and development. Natural product chemistry has been an effective strategy for the identification of bioactive lead candidates. An interesting group of natural products are the diterpenes, as they possess a plethora of biological activities and display many pharmacological properties with therapeutic potential [9,10,11,12,13,14]. However, no report has been issued on the anti-inflammatory effects of labdane diterpenes isolated from the rhizomes of *Solidago chilensis*, a plant traditionally used to treat symptomatologies related to inflammation [15].

Therefore, as part of our ongoing screening program to evaluate the potential of diterpenes as a source of novel bioactive compounds in inflammation-based diseases, a series of diterpenes were semisynthesized from the natural diterpene solidagenone. Herein, we report the preparation and anti-inflammatory effects of these solidagenone derivatives (**2**−**10)** in peritoneal macrophages.

Among the derivatives evaluated, solidagenone derivative **5** exhibited significant anti-inflammatory activity in TLR-activated peritoneal macrophages. This derivative suppressed the production of proinflammatory mediators via inhibition of the NOS-2 and COX-2 enzymes, as well as cytokines. These effects were accompanied by the downregulation of NF-κB activation and mitogen-activated protein kinase (MAPK) phosphorylation.

## 2. Results and Discussion

**Scheme 1:**
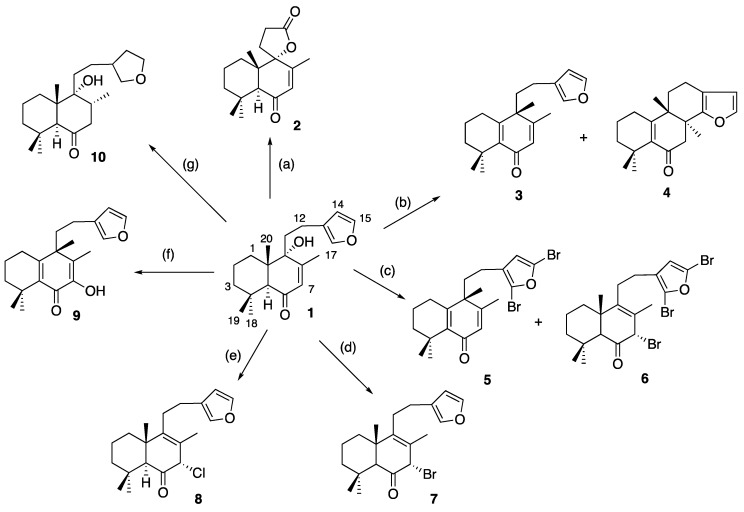
Solidagenone derivatives. Reagents and conditions: (a) NaBH_4_, ethanol, rt; (b) AcCl, rt; (c) Br_2_, dry DCM, rt; (d) BBr_3_, dry DCM, 0 °C; (e) SOCl_2_, dry DCM, reflux; (f) NaH; dry THF, reflux; (g) H_2_, 10% Pd/C, rt.

Several modifications were carried out on the diterpene solidagenone (**1**), aiming to obtain new derivatives and to evaluate the role of the different functional groups and moieties present in the structure of **1** (Scheme 1) in its anti-inflammatory activity.

**Figure 1:**
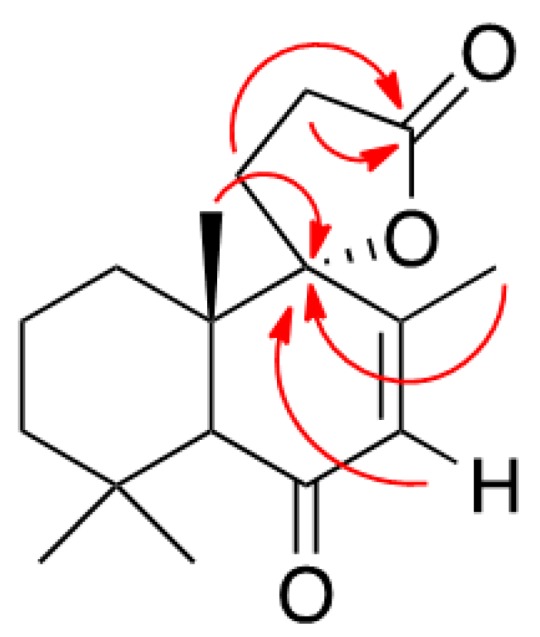
Key HMBC correlations for **2**.

Thus, the treatment of solidagenone with NaBH_4_ yielded the unexpected spirolactone (**2**). Compound **2** has three fewer carbons than **1**, and it is probably formed by rupture of the furan ring followed by reductive cyclization. The existence of the lactone moiety was supported by the presence of a carbonyl group at 176.0 ppm, a quaternary oxygenated carbon at δ 90.0, and two methylene carbons at δ 30.9 and 23.9, respectively. The HSQC and HMBC (Figure 1) correlations observed for (**2**) in the corresponding spectra ratified its structure.

**Scheme 2:**
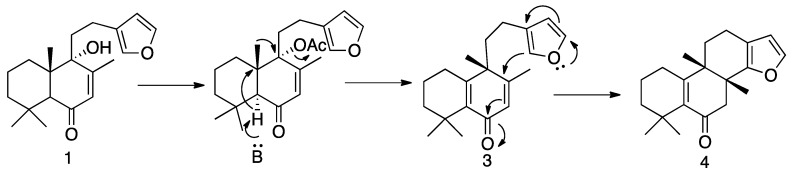
Plausible formation of compounds **3** and **4**.

When **1** was treated with AcCl/Py, the two compounds (**3**) and (**4**) were formed. A plausible mechanism of formation involves the acetylation of the hydroxyl group and elimination of the corresponding acetyl group via 1,2-methyl shift to afford (**3**). An intramolecular Michael addition of the furan ring on the α,β-unsaturated carbonyl system could explain the formation of the tetracyclic compound (**4**) (Scheme 2).

**Figure 2:**
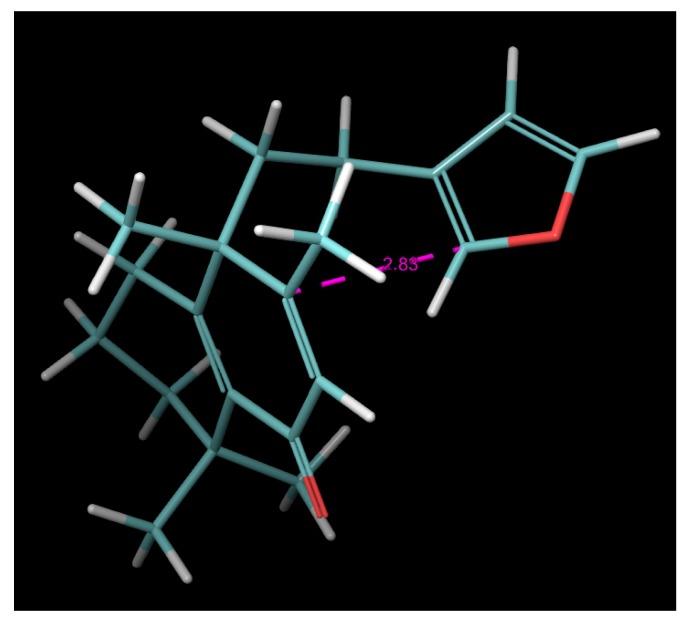
Representative minimum-energy conformer of compound **3**.

The minimum-energy conformers for compound **3** were analyzed in order to corroborate this plausible mechanism. One of them (Figure 2) showed an appropriate distance (2.83 Å) and relative spatial position between the carbons C-8 and C-16 to support the intramolecular ring-closing reaction [16]. The *syn* orientation of the methyl groups on the new formed ring was ratified by the NOEs detected between the methyl groups at C-8 and C-9, respectively.

When **1** was reacted with 2 equiv. of Br_2_/DCM, the brominated compounds (**5**) and (**6**) were formed. The regiosubstitution of the furan ring was determined by an HMBC experiment. Thus, the following HMBC correlations: H-14 with C-13, C-15, and C-16; and H-12 with C-13, C-14, and C-16 located the two bromine atoms at the carbons C-15 and C-16. The third bromine was located at C-7 in compound **6**, since a singlet was observed at δ 4.37, which showed HMBC correlations with the carbonyl carbon at δ 203.3 (C-6) and with the olefinic carbons C-8 and C-9. The relative orientation was determined as β on the basis of the ROEs detected in the ROESY spectrum. The reaction of **1** with BBr_3_ led to the monobrominated derivative (**7**), which showed similar NMR data to compound **6**, but corresponding to the furan ring. A similar chlorine derivative (**8**) was obtained when **1** was reacted with thionyl chloride in DCM. The treatment of **1** with NaH in THF led to derivative (**9**). The hydrogenation of **1** yielded the corresponding derivative (**10**) with the furan ring reduced to tetrahydrofuran. The orientation α of the methyl group at C-8 was established on the basis of the NOE effect between the multiplet at δ 2.34 (1H, H-8) and the singlet at δ 1.03 (3H, H-20).

### 2.1. Effects of Solidagenone Derivatives on Inflammatory Responses

**Figure 3:**
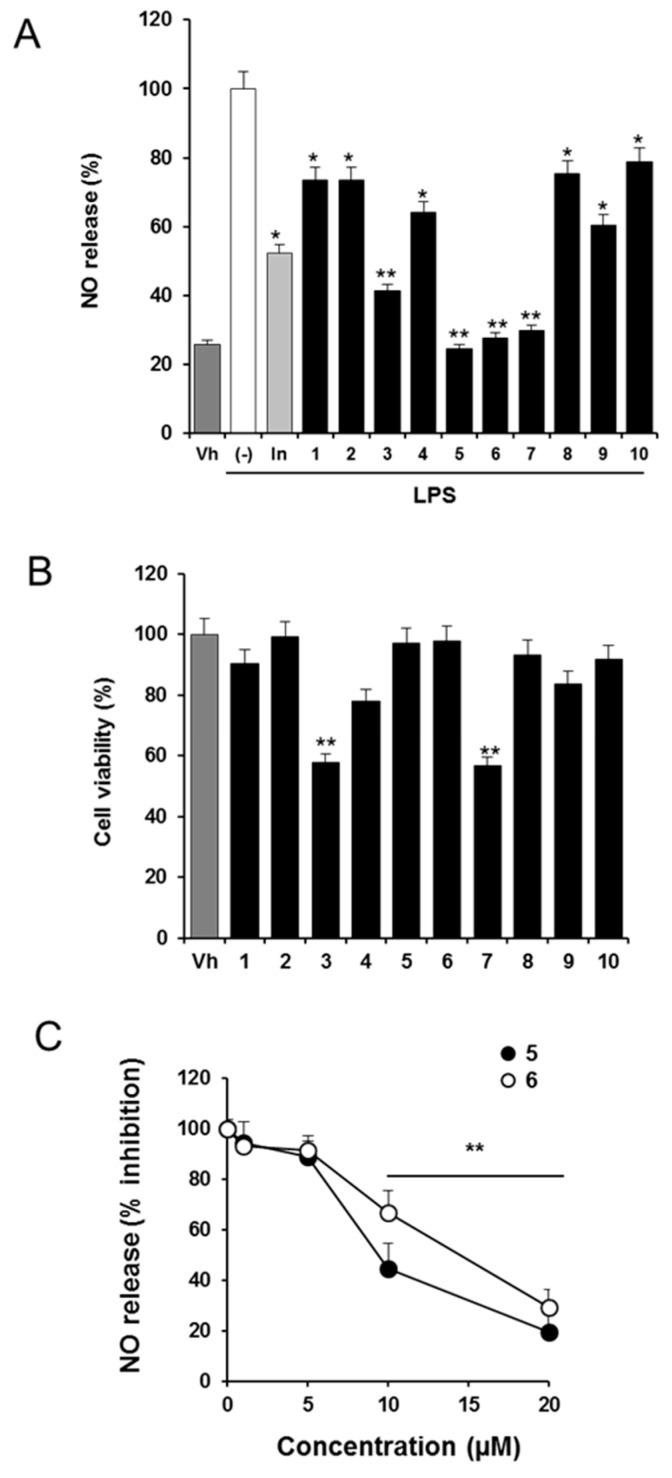
Effects of solidagenone derivatives on nitric oxide (NO) release and cell viability. (**A**) Peritoneal macrophages were treated with vehicle (Vh), lipopolysaccharide (LPS) (200 ng/mL), LPS and 10 µM of indomethacin (In), or LPS and 20 µM of solidagenone derivatives (**1**–**10**) for 24 h. The accumulation of nitrite in the culture medium was measured with the Griess method and expressed as the percentage of NO release compared to LPS treatment. (**B**) Peritoneal macrophages were treated with vehicle (Vh) or 20 µM of solidagenone derivatives (**1**–**10**) for 24 h. Cell viability was determined by MTT assay. (**C**) Dose-dependent effects of compounds **5** and **6** (5–20 μM) on NO release. Experiments were carried out in triplicate and results are the means ± SD of three independent experiments. * *p* < 0.05 and ** *p* < 0.01 with respect to the LPS treatment.

To evaluate whether these diterpenes (**1**–**10**) regulate the inflammatory response, we investigated the anti-inflammatory effects and underlying mechanisms of action of these derivatives in peritoneal macrophages. We first identified compounds which can inhibit the inflammatory response induced by bacterial lipopolysaccharide (LPS), a well-characterized ligand of TLR4 in murine peritoneal macrophages. For this, cells were preincubated with diterpenes for 30 min before their stimulation with LPS (200 ng/mL), and NO production was assayed. Initial testing of the compounds at a fixed concentration of 20 µM revealed that all the derivatives exhibited significant inhibitory effects on NO release. Four compounds (**3**, **5**, **6**, and **7**) were able to inhibit NO release by more than 50% (Figure 3A). Moreover, they also displayed higher inhibitory activity than indomethacin at 10 µM, used as a reference anti-inflammatory drug.

To discard the possibility that the inhibitory effect on NO release was due to cytotoxicity, we analyzed the percentage of cell viability by MTT (3-(4,5-dimethylthiazol-2-yl)-2,5-diphenyltetrazolium bromide) assay. As shown in Figure 3B, compounds **3** and **7** significantly reduced cell viability, whereas the rest of compounds tested were noncytotoxic, as cell viability was not significantly affected by compounds in the 10–50 μM range where the anti-inflammatory activity was studied (data not shown).

From the obtained results, some structure–activity relationships were outlined. It was thus found that the presence of bromine groups is important for the activity in the bicyclic derivatives. If we compare the inhibition of NO release of compound **4** with that of compound **5**, the presence of bromines in the furan ring is associated with a lower percentage of NO release. The replacement of a bromine group at C-7 (**7**) by a chlorine group (**8**) led to a lower inhibition of NO production.

In view of these data, compounds **5** and **6** were selected for further evaluation. Studies of the dose–response effects of compounds **5** and **6** on NO release showed NO inhibitory effects with IC_50_ values of 9.1 ± 1.51 and 14.52 ± 1.03 µM, respectively (Figure 3C).

LPS has been the most studied TLR4 ligand. The binding of LPS to TLR4 on macrophages activates the transcription factor NF-κB, a key step in the onset of the inflammatory response. NF-κB regulates the expression of proinflammatory mediators and enzymes such as NOS-2 and COX-2. To further analyze the signaling pathways modulated by the two selected compounds **5** and **6**, we studied the activation of the proinflammatory genes NOS-2 and COX-2.

**Figure 4:**
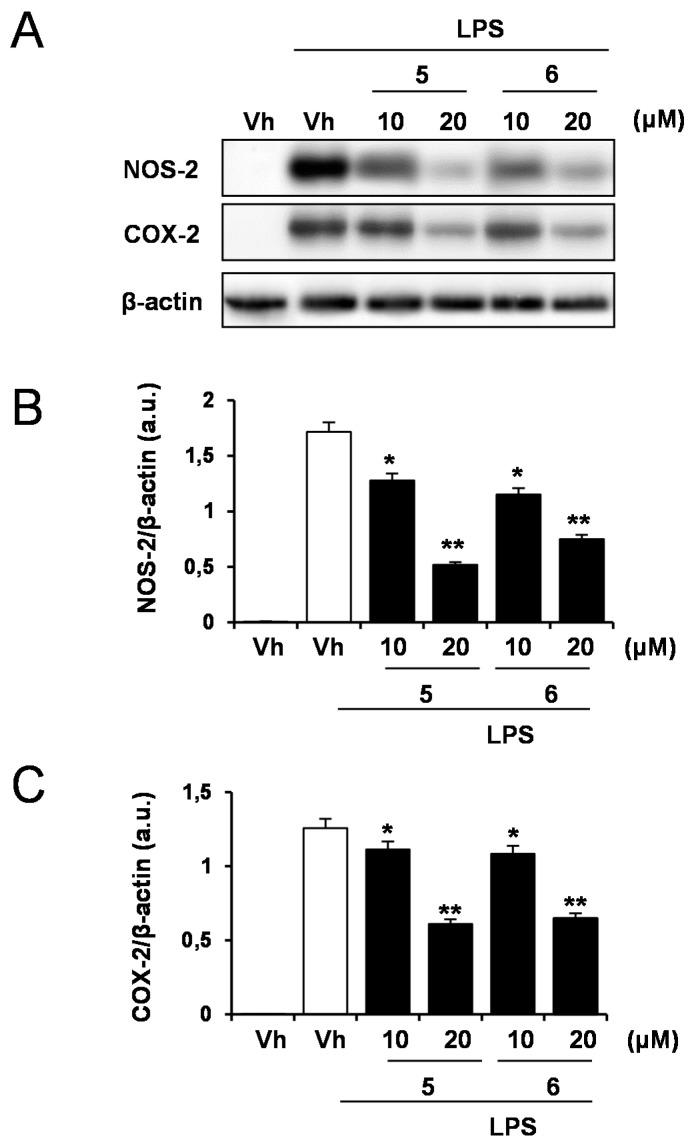
NOS-2 and COX-2 expression is inhibited by solidagenone derivatives. (**A**) Macrophages were preincubated for 30 min with derivatives **5** and **6** (10 and 20 μM) followed by stimulation with 200 ng/mL LPS for 24 h. NOS-2 and COX-2 proteins were detected by Western blot. β-actin content was used as a loading control. The Western blot in (**A**) shows a representative experiment. (**B**–**C**) Densitometry analysis of NOS-2 (**B**) and COX-2 (**C**) expression was performed. Results show the mean ± SD of three independent Western blot experiments and are expressed as the percentage of relative signal intensity with respect to the LPS condition. * *p* < 0.05, ** *p* < 0.01, with respect to the LPS condition.

In unstimulated peritoneal macrophages, the levels of NOS-2 and COX-2 proteins were undetectable. As expected, LPS treatment induced the activation of both proteins. However, when macrophages were preincubated with compounds **5** and **6** and stimulated with LPS for 24 h, a significant inhibition of the protein expression of NOS-2 and COX-2 was observed (Figure 4). Although similar inhibitory effects on NOS-2 and COX-2 expression were observed for both solidagenone derivatives, compound **5** exhibited the lowest IC_50_ value, being selected for further studies.

**Figure 5:**
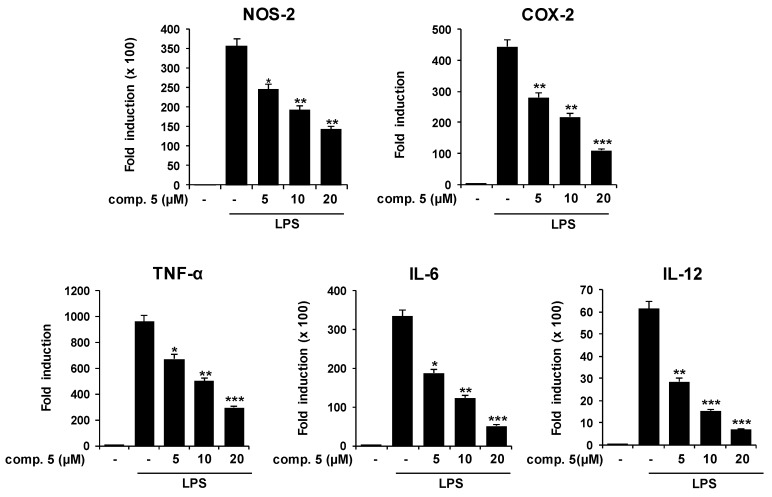
Diterpene **5** inhibits expression of inflammatory genes in a dose-dependent manner. Macrophages were preincubated for 30 min with compound 5 (5, 10, and 20 μM) followed by stimulation with 200 ng/mL LPS for 4 h. Relative expression of NOS-2, COX-2, TNF-α, IL-6, and IL-12 mRNA was determined by real-time quantitative PCR. Results are the means ± SD of three independent experiments carried out in triplicate. * *p* < 0.05, ** *p* < 0.01, and *** *p* < 0.001 vs. the LPS condition.

Analysis of NOS-2 and COX-2 mRNA by real-time quantitative PCR revealed that compound **5** inhibited LPS-induced expression of both enzymes, acting at the transcriptional level (Figure 5). In order to explore whether other inflammatory mediators such as cytokines were affected by treatment with compound **5**, we next analyzed levels of tumor necrosis factor-α (TNF-α), interleukin (IL)-6, and IL-12. All cytokines were downregulated at the mRNA level in the presence of compound **5** after stimulation with LPS (Figure 5). Interestingly, these data demonstrated that this derivative has a broad effect on the transcription of proinflammatory genes associated with the LPS-induced response.

**Figure 6:**
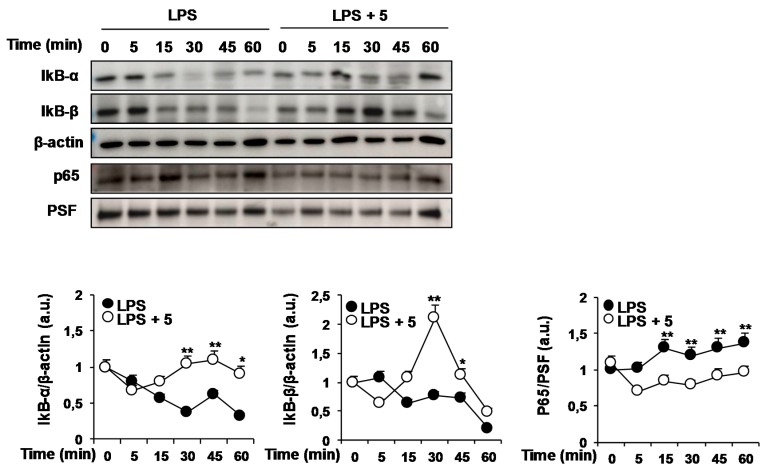
Diterpene **5** inhibits NF-κB activation in LPS-stimulated murine macrophages. Macrophages were pretreated for 30 min with compound **5** (20 μM) and then activated for the indicated times (5, 15, 30, 45, and 60 min) with 200 ng/mL LPS. The levels of proteins IκBα, IκBβ, and p65 were determined by Western blot. β-actin and PSF were used as loading controls. A representative experiment is shown. Densitometry analysis shows the mean ± SD of three independent Western blot experiments. * *p* < 0.05, ** *p* < 0.01 vs. LPS.

**Figure 7:**
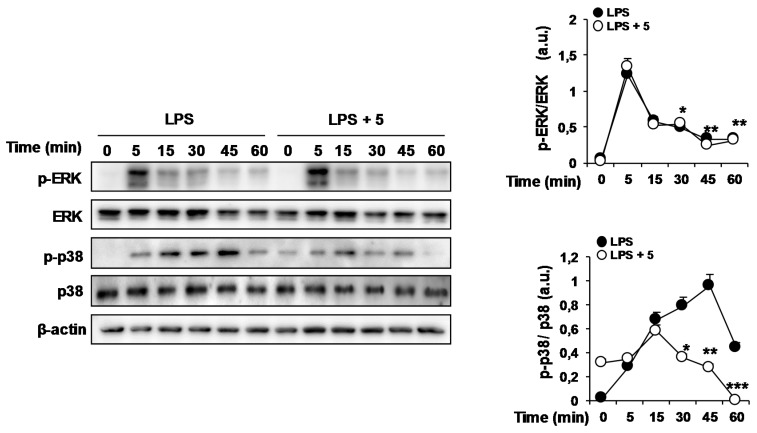
Regulation of MAPK activities by diterpene **5**. Macrophages were pretreated for 30 min with compound **5** (20 μM) and then activated for the indicated times (5, 15, 30, 45, and 60 min) with 200 ng/mL LPS. Levels of phospho- and total MAPKs (ERK and p38) were determined by Western blot. A representative experiment is shown. Densitometry analysis shows the mean ± SD of three independent Western blot experiments. * *p* < 0.05, ** *p* < 0.01, and *** *p* < 0.001 vs, LPS.

TLR ligands activate various downstream intracellular signaling cascades, such as the NF-κB and MAPK pathways. NF-κB activation requires the phosphorylation and degradation of inhibitory kappa B (IκB) proteins, leading to the translocation of p65/p50 heterodimers to the nucleus. Interestingly, this transcription factor has been a common target in the mechanism of action of some diterpenes [9,14,17,18,19]. The activation of MAPK cascades, including extracellular-signal regulated kinase 1/2 (ERK1/2), c-Jun-N-terminal kinase (JNK), and p38 MAPK, is another event triggered by TLR ligands in macrophages [20,21,22], which also plays important roles in the induction of proinflammatory genes. Thus, we next evaluated whether the anti-inflammatory effect of compound **5** could be attributed to inhibitory effects on these pathways. First, we examined its effects on the levels of cytosolic IκBα and IκBβ proteins in LPS-activated macrophages. As shown in Figure 6, degradation of IκBα and IκBβ were impaired in activated cells pretreated with compound **5**. Furthermore, a reduced accumulation of the NF-κB p65 subunit in the nucleus was observed after treatment with diterpene **5**. In addition, compound **5** also inhibited p38 phosphorylation (Figure 7), whereas the activation of other MAPKs (ERK1/2 or JNK) was not affected (Figure 7 and data not shown). These data suggest that the diterpene **5** affects proinflammatory signaling mainly through regulation of NF-κB and p38 activation.

### 2.2. Effects of Compound ***5*** in Response to Different TLR Ligands

Pharmacological regulation of TLR signaling is a promising therapeutic strategy for various inflammatory and autoimmune diseases [23,24,25]. Other TLR ligands, including TLR2 and TLR3, also induce inflammatory responses in macrophages. In order to determine if compound **5** exhibits inhibitory effects against other TLRs, we examined the response of macrophages to the TLR3 ligand polyriboinosinic–polyribocytidylic acid (polyI:C) and to the TLR2 ligand lipoteichoic acid (LTA).

**Figure 8:**
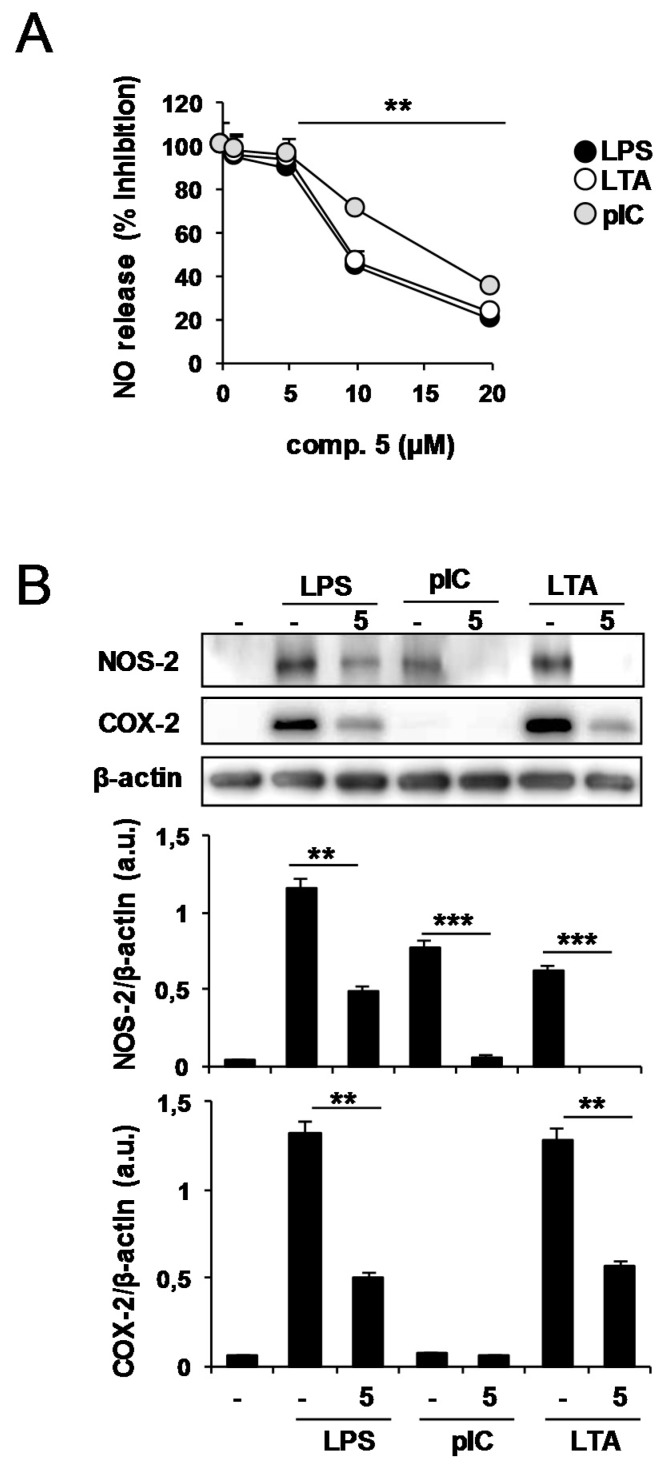
Compound **5** inhibits NO release and gene expression mediated by different Toll-like receptors (TLRs). (**A**) Peritoneal macrophages were incubated with diterpene **5** (5–20 µM) or vehicle for 30 min, followed by stimulation with (TLR ligands): LPS 200 ng/mL (TLR4 ligand), polyriboinosinic–polyribocytidylic acid (poly I:C) 20 µg/mL (TLR3 ligand), or lipoteichoic acid (LTA) 10 µg/mL (TLR2 ligand) for 24 h. The accumulation of nitrite in the culture medium was measured with the Griess reagent. Experiments were carried out in triplicate and results show the mean ± SD of three independent experiments. (**B**) Peritoneal macrophages were incubated with diterpene **5** (20 μM) or vehicle for 30 min, followed by stimulation with TLR ligands for 20 h. NOS-2 and COX-2 proteins were detected by Western blot and β-actin was used as a loading control. A representative experiment of three is shown. Densitometry analysis shows the mean ± SD of three independent Western blot experiments. ** *p* < 0.01, *** *p* < 0.001.

The accumulation of the proinflammatory mediator NO in the culture medium was also dose-dependently (5–20 µM) inhibited, regardless of ligand treatment (Figure 8A). The dose–response curves for all three ligands showed comparable inhibition. As shown in Figure 8B, LPS, polyI:C, and LTA promoted the expression of NOS-2 in macrophages, an effect that was attenuated in the presence of compound **5**. COX-2 expression induced by LPS and LTA was also inhibited by compound **5**. These results suggest that this diterpene has a broad suppressive effect on TLR-mediated inflammatory responses. Considering the pathological role of TLRs in inflammatory diseases, inhibitors targeting TLR signaling may emerge as novel therapeutics to treat these disorders [24].

### 2.3. Molecular Docking Studies

In order to propose a mode of action of the significant anti-inflammatory effects of the solidagenone derivatives **5** and **6**, we carried out a molecular docking study using the Glide software [26] on reported the crystal structure of human TLR4 in complex with MD-2 and LPS (PDB 4G8A). Like the extracellular domains of other TLRs, TLR4 contains leucine-rich repeats and adopts a characteristic horseshoe-like shape. MD-2 is noncovalently bound to the side of the horseshoe ring and also directly interfaces with the ligand. MD-2 has a β-cup fold structure composed of two antiparallel β-sheets, forming a large hydrophobic pocket for ligand binding. The molecular docking study is a well-known and effective method to predict the binding mode of the ligand to its receptors. Hence, in order to explore the binding mode of solidagenone derivatives **5** and **6**, we simulated docking of the compounds into the large hydrophobic binding pocket of MD-2 to see if these compounds could interact with this target, and consequently, to understand the possible binding mode and key active site interactions. An analysis of the docking results showed that the compounds fit very well and could be inserted into the large hydrophobic binding pocket of MD-2 and easily occupy a large portion of the LPS binding site. In addition, these docking results strongly suggested that the solidagenone derivatives **5** and **6** share a common binding mode into the hydrophobic binding pocket of MD-2. The best docking scores were found in the range from −6.83 to −8.51 kcal mol^−1^. According to the predicted binding modes, the most active compounds showed a π–π stacking interaction between the furan ring present in these compounds and some amino acid residues. Hydrophobic interactions therefore probably play a dominant role in the interaction. In the favored docking conformation, many hydrophobic side-chain residues of the MD-2 pocket are in close proximity to the solidagenone derivatives.

**Figure 9:**
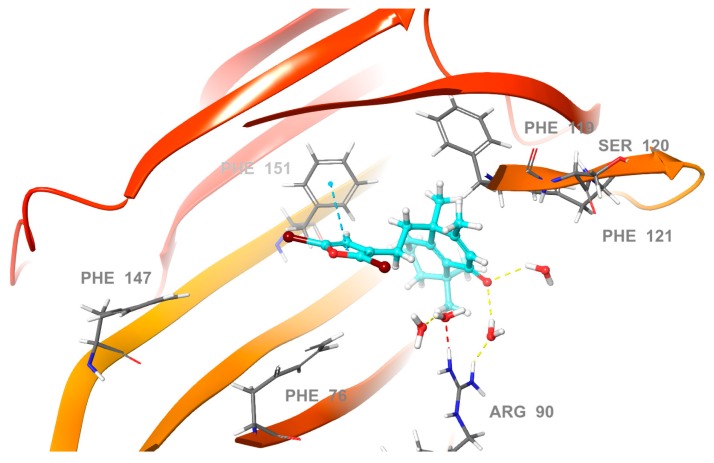
Docking model and key interactions of compound **5** into the TLR4 active site (PDB 4G8A).

In the predicted pose of the most active compound (**5**), the furan ring showed a key π–π interaction with Phe 76 (Figure 9). There are also multiple potential hydrophobic interactions with MD-2, involving residues such as Leu 61, Phe 104, Ile 94, Phe 151, Phe 147, Tyr 102, Val 135, Ile 46, Ile 63, and Ile 117 (Figure 9). Taken together, such interactions of the compound with MD-2 suggest that the binding of **5** forms a bulky hindrance that blocks the interaction of LPS with MD-2.

### 2.4. Molecular Dynamics (MD) Simulation

The docking protocols can be combined with accurate molecular dynamics (MD) simulation techniques to predict more reliable protein–ligand complex structures and confirm system stability. We explored the stability of the best docking pose of **5** with molecular dynamics (MD) simulation, and the protein–ligand interactions were investigated throughout the course of the MD simulations. The simulations provide minute detail about the movement of every particle/atom over time. This will help in answering the questions arising about the deviation and fluctuation patterns of the proteins.

**Figure 10:**
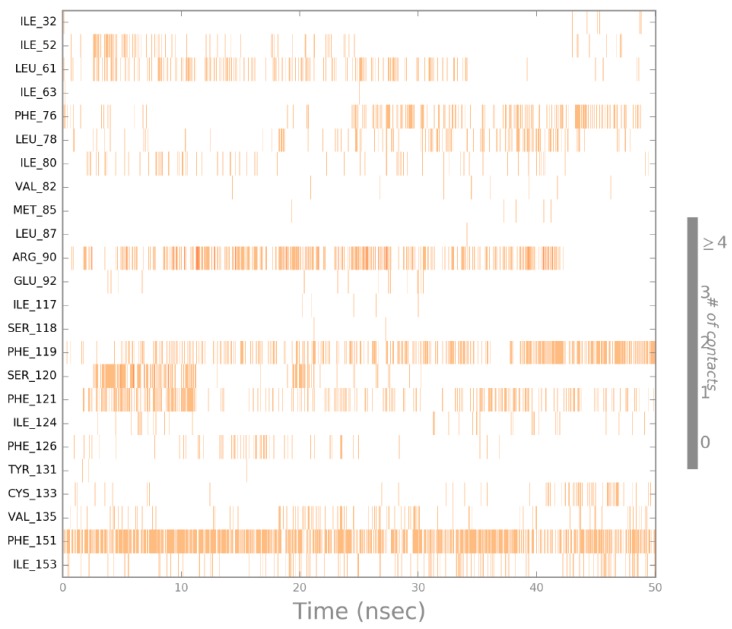
Interaction diagram for compound **5** and MD-2 complex obtained in molecular dynamics simulation studies.

The MD simulation of the energy-minimized docked complex with the best docking scores of compound **5** with MD-2 was performed over 50 ns in an explicitly aqueous solution environment employing the Desmond Software (which was developed at D. E. Shaw Research to perform high-speed molecular dynamics simulations of biological systems), the OPL-2005 force field, and the TIP3P solvent model. Molecular dynamic simulations were performed to confirm the orientation of compound **5** in the binding site. The MD simulation results indicated that the compound does not leave the binding cavity of the protein and remains in a similar orientation during the entire simulation (Figure 10).

**Figure 11:**
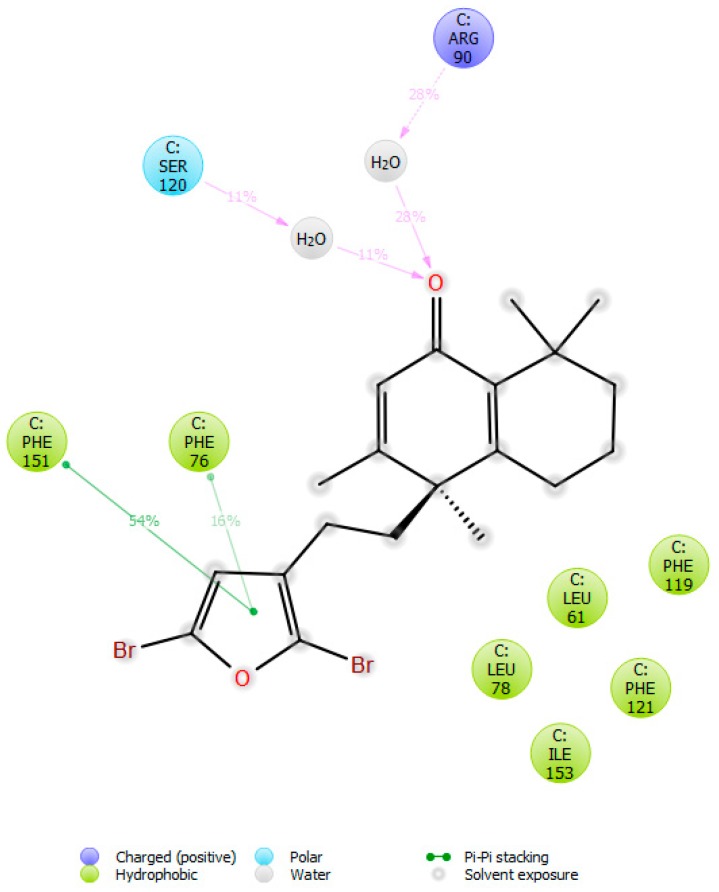
Interactions of compound **5** with the key amino acid residues at the hydrophobic binding pocket of MD-2 (docking score: −8.51 kcal mol^−1^). The colors indicate the residue type: green are lipophilic residues; blue are polar residues; purple are basic residues. Ligand atoms that are exposed to the solvent are marked with grey spheres. C: means MD2 region of TLR4.

In the graphical snapshot of the production phase, it was revealed that compound **5** efficiently interacted through water bridges to form two H-bond interactions between the carbonyl group present in the solidagenone scaffold and the critical polar amino acid residues Ser 120 and Arg 90. It indicated that compound **5** is additionally stabilized by hydrogen bonds, which may explain the potency of this derivative. However, the interaction with Ser 120 is less significant, since it was only observed for about 11% of the simulation time, while the interaction with Arg 90 occurred for more than 28% of the simulation time in the selected trajectory (Figure 11).

**Figure 12:**
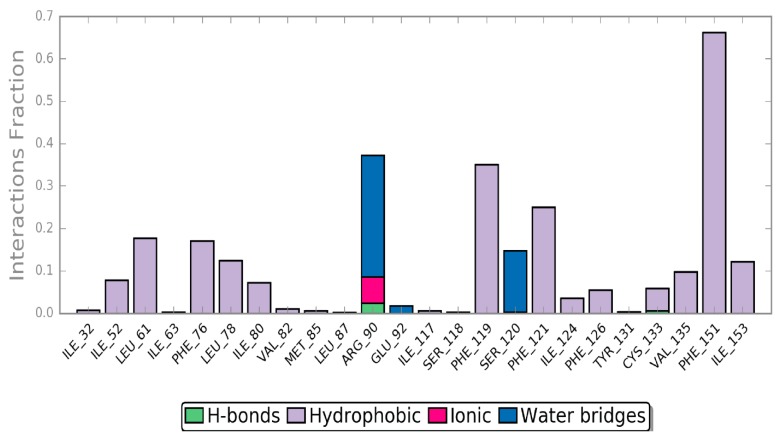
The stacked bar charts of MD-2 interaction with compound **5**.

In addition, it could also be observed that compound **5** interacted with other essential amino acid residues such as Phe 119, Phe 121, and Phe 151, which are part of the large hydrophobic pocket by means of hydrophobic interactions. These interactions of the protein with the ligand could be observed as interaction fractions throughout the molecular dynamics simulation (Figure 12). The stacked bar charts are normalized over the course of the trajectory: for example, a value of 0.7 suggests that the specific interaction is maintained for 70% of the simulation time. This allowed us to establish the role of each of the particular bonds with the amino acid residues responsible for the stabilization of the complex. This model and subsequent dynamical observation can help in explaining the activity of compound **5** confirming the potential of solidagenone derivatives as promising candidates for TLR4 inhibitors.

## 3. Materials and Methods

### 3.1. Chemistry

#### 3.1.1. General Methods

NMR spectra were recorded in CDCl_3_ or at 400 MHz for ^1^H NMR and 100 MHz for ^13^C NMR. Chemical shifts are given in (δ) parts per million and coupling constants (*J*) in hertz (Hz). ^1^H and ^13^C spectra were referenced using the solvent signal as the internal standard. HREIMS were recorded using a high-resolution magnetic trisector (EBE) mass analyzer. Analytical thin-layer chromatography plates used were POLYGRAM-SILG/UV. Preparative thin-layer chromatography was carried out with Analtech GF plates (20 × 20 cm, 1000 microns) using appropriate mixtures of ethyl acetate and hexanes. All solvents and reagents were purified by standard techniques as reported previously [27] or used as supplied from commercial sources. Solidagenone (**1**) was isolated from the air-dried rhizomes of *Solidago chilensis* Meyen (Asteraceae) as previously reported [28].

#### 3.1.2. Solidagenone (**1**)

^1^HNMR (400 MHz) δ 1.00 (3H, s, H-20), 1.14 (3H, s, H-18), 1.16 (1H, m, H-3a), 1.19 (3H, s, H-20), 1.34 (1H, m, H-3b), 1.52 (2H, m, H-1), 1.57 (2H, m, H-2), 1.77 (1H, m, H-11a), 1,90 (1H, m, H-11b), 2.01 (3H, s, H-17), 2.65 (2H, m, H-12), 2.70 (1H, s, H-5), 5.70 (1H, s, H-7), 6.30 (1H, s, H-14), 7.25 (1H, s, H-16), 7.37 (1H, s, H-15); ^13^C-NMR (100 MHz) δ 200.1 (C-6), 155.6 (C-8), 143.1 (C-15), 138.5 (C-16), 129.2 (C-7), 125.1 (C-13), 110.7 (C-14), 76.5 (C-9), 55.8 (C-5), 46.4 (C-10), 42.6 (C-3), 33.8 (C-19), 33.2 (C-11), 32.2 (C-4), 31.6 (C-1), 21.7 (C-18), 21.2 (C-12), 20.0 (C-20), 18.3 (C-17), 17.8 (C-2); HR-ESMS *m*/*z* 316.2047 (calculated for C_20_H_28_O_3_, 316.2038).

#### 3.1.3. Preparation of Solidagenone Derivative (**2**)

To a solution of 55 mg (0.17 mmol) of **1** in 2 mL ethanol, a solution of 15 mg (2 equiv.) of NaBH_4_ in 2 mL of EtOH was added dropwise at rt. After 2 h stirring, the mixture was acidified with 10% HCl and extracted with DCM. The organic phase was dried with MgSO_4_, filtered, and concentrated. The residue was purified by preparative TLC using hexane/EtOAc 4:1 as the eluent to yield 8 mg (17% yield) of **2**.

^1^H-NMR (400 MHz) δ 5.76 (1H, s, H-7), 2.68 (1H, m, H-12), 2.64 (1H, s, H-5), 2.58 (1H, m, H-12), 2.28 (1H, m, H-11), 2.14 (1H, m, H-11), 1.84 (3H, s, H-14), 1.52 (3H, m, H-1, H-2), 1.45 (1H, m, H-1), 1.31 (2H, m, H-3), 1.16 (6H, s, H-15, H-16), 0.96 (3H, s, H-17); ^13^C-NMR (100 MHz) δ 198.9 (C-6), 176.0 (C-13), 149.3 (C-8), 130.9 (C-7), 90.0 (C-9), 56.5 (C-5), 44.5 (C-10), 42.1 (C-3), 33.1 (C-16), 32.2 (C-4), 31.5 (C-1), 30.9 (C-12), 23.9 (C-11), 21.6 (C-15), 18.8 (C-17), 18.1 (C-2), 17.5 (C-14); HR-ESMS *m*/*z* 299.1623 (calculated for C_17_H_24_O_3_Na, 299.1623).

#### 3.1.4. Preparation of Solidagenone Derivatives (**3**) and (**4**)

A solution of 50 mg (0.16 mmol) of **1** in 2 mL of *N*,*N*-dimethylaniline was treated with an excess of AcCl (2 mL) and stirred at rt for 2 h. Then, ice was added and the reaction mixture was extracted with DCM. The solvent was evaporated and the residue was purified by preparative TLC, using hexane/EtOAc 4:1 as the eluent, to obtain 7 mg (15%) of compound **3** and 17 mg (37%) of compound **4**.

*Compound***3**: ^1^H-NMR (400 MHz) δ 7.33 (1H, br s, H-15), 7.14 (1H, s, H-16), 6.18 (1H, brs, H-14), 6.14 (1H, s, H-7), 2.34 (1H, m, H-1), 2.20 (1H, m, H-1), 1.95 (3H, s, H-17), 1.92 (2H, m, H-12), 1.85 (2H, m, H-3), 1.63 (2H, m. H-2), 1.47 (2H, m, H-11), 1.32 (3H, s, H-19), 1.30 (3H, s, H-18), 1.20 (3H, s, H-20); ^13^C-NMR (100 MHz) δ 185.8 (C-6), 159.8 (C-8), 158.1 (C-10), 142.8 (C-15), 141.1 (C-5), 138.5 (C-16), 130.6 (C-7), 124.2 (C-13), 110.6 (C-14), 46.4 (C-9), 41.5 (C-11), 37.4 (C-3), 33.7 (C-4), 28.4 (C-19), 28.0 (C-18), 27.4 (C-1), 25.4 (C-20), 19.9 (C-12), 19.0 (C-2), 18.9 (C-17); HR-ESIMS *m*/*z* 321.1829 (calculated for C_20_H_26_O_2_Na, 321.1831).

*Compound***4**: ^1^H-NMR (400 MHz) δ 7.21 (1H, br s, H-15), 6.10 (1H, br s, H-14), 2.73 (1H, d, *J* = 16.1 Hz, H-7), 2.52 (1H, d, *J* = 16.1 Hz, H-7), 2.42 (1H, m, H-1), 2.32 (3H, m, H-1, H-12), 1.95 (1H, m, H-11), 1.84 (1H, m, H-11), 1.58 (2H, m, H-2), 1.42 (2H, m, H-3), 1.24 (3H, s, H-19), 1.21 (3H, s, H-18), 1.14 (3H, s, H-17), 1.06 (3H, s, H-20); ^13^C-NMR (100 MHz) δ 196.5 (C-6), 159.2 (C-10), 154.6 (C-5), 154.6 (C-16), 141.1 (C-15), 114.5 (C-13), 109.6 (C-14), 43.5 (C-9), 41.5 (C-3), 41.4 (C-7), 39.7 (C-8), 33.6 (C-4), 30.4 (C-11), 28.8 (C-19), 28.6 (C-18), 28.5 (C-1), 28.5 (C-20), 19.4 (C-12), 19.3 (C-2), 19.2 (C-17); HR-ESIMS *m*/*z* 321.1833 (calculated for C_20_H_26_O_2_Na, 321.1831).

#### 3.1.5. Preparation of Solidagenone Derivatives (**5**) and (**6**)

A solution of 100 mg (0.32 mmol) of **1** in 3 mL of dry DCM was stirred with 32 μL (2 equiv.) of Br_2_ at rt for 15 h. Then, the solvent was evaporated and the residue was purified by preparative TLC, using hexane/EtOAc 9:1 as the eluent. Two products were obtained: 22 mg (15%) of **5** and 17 mg (11%) of **6**.

Compound **5**: ^1^H-NMR (400 MHz) δ 6.15 (2H, brs, H-7, H-14), 2.32 (1H, m, H-1), 2.22 (1H, m, H-1), 1.96 (3H, s, H-17), 1.82 (4H, m, H-3, H-12), 1.67 (2H, m, H-2), 1.51 (2H, m, H-11), 1.32 (3H, s, H-19), 1.31 (3H, s, H-18), 1.23 (3H, s, H-20); ^13^C-NMR (100 MHz) δ 185.7 (C-6), 159.2 (C-8), 157.7 (C-10), 141.4 (C-5), 130.7 (C-7), 126.2 (C-15), 121.6 (C-13), 119.6 (C-16), 114.5 (C-14), 46.2 (C-9), 41.5 (C-11), 36.8 (C-3), 33.7 (C-4), 28.5 (C-19), 28.0 (C-18), 27.4 (C-1), 25.1 (C-20), 20.6 (C-12), 19.0 (C-2), 18.9 (C-17); HR-ESIMS *m*/*z* 477.0034 (calculated for C_20_H_24_O_2_Br_2_Na, 477.0041).

Compound **6**: ^1^H-NMR (400 MHz) δ 6.28 (1H, s, H-14), 4.37 (1H, s, H-7), 3.22 (1H, s, H-5), 2.45 (2H, t, *J =* 8.7 Hz, H-12), 2.28 (1H, m, H-1), 2.18 (1H, m, H-1), 1.95 (1H, br d, *J =* 12.2 Hz, H-11), 1.86 (3H, s, H-17), 1.69 (2H, m, H-2), 1.56 (1H, m, H-11), 1.42 (1H, br d, *J =* 13.2 Hz, H-3), 1.32 (3H, s, H-18), 1.17 (1H, m, H-3), 1.04 (3H, s, H-19), 0.95 (3H, s, H-20); ^13^C-NMR (100 MHz) δ 203.3 (C-6), 147.7 (C-8), 126.4 (C-9), 126.3 (C-13), 121.8 (C-15), 119.6 (C-16), 114.4 (C-14), 57.7 (C-5), 54.9 (C-7), 46.3 (C-10), 41.8 (C-3), 36.1 (C-11), 31.9 (C-4), 31.6 (C-19), 28.8 (C-1), 25.0 (C-12), 22.0 (C-18), 21.5 (C-20), 18.6 (C-2), 17.4 (C-17); HR-ESIMS *m*/*z* 556.9216 (calculated for C_20_H_25_O_2_Br_3_Na, 556.9302).

#### 3.1.6. Preparation of Solidagenone Derivative (**7**)

A solution of 100 mg (0.32 mmol) of **1** in 5 mL of dry DCM at 0 °C was treated with 0.63 mL (2 equiv.) of a 1 M solution of BBr_3_ in DCM. The mixture was stirred for 4 h. Then, the solvent was evaporated and the residue purified by preparative TLC using hexane/EtOAc 9:1 as the eluent to yield 24 mg (21%) of compound **7**. ^1^H-NMR (400 MHz) δ 7.37 (1H, s, H-15), 7.26 (1H, s, H-16), 6.30 (1H, br s, H-14), 4.38 (1H, s, H-7), 3.23 (1H, s, H-5), 2.55 (2H, t, J *=* 8.6 Hz, H-12), 2.35 (1H, m, H-1), 2.23 (1H, m, H-1), 1.91 (1H, brd, *J* = 16.7 Hz, H-11), 1.72 (3H, s, H-17), 1.65 (2H, m, H-2), 1.49 (1H, m, H-11), 1.41 (1H, brd, *J* = 12.9 Hz, H-3), 1.32 (3H, s, H-18), 1.17 (1H, m, H-3), 1.01 (3H, s, H-19), 0.96 (3H, s, H-20); ^13^C-NMR (100 MHz) δ 203.5 (C-6), 148.8 (C-8), 142.9 (C-15), 138.5 (C-16), 125.7 (C-9), 124.4 (C-13), 110.6 (C-14), 57.8 (C-5), 55.3 (C-7), 46.3 (C-10), 41.8 (C-3), 36.1 (C-11), 31.9 (C-4), 31.7 (C-19), 29.6 (C-1), 24.6 (C-12), 22.0 (C-18), 21.5 (C-20), 18.6 (C-2), 17.4 (C-17); HR-EIMS *m*/*z* 378.1122 (calculated for C_20_H_27_O_2_Br, 378.1195).

#### 3.1.7. Preparation of Solidagenone Derivative (**8**)

To a solution of 100 mg (0.316 mmol) of **1** in 5 mL of dry DCM, 92 μL (4 equiv.) of SOCl_2_ was added under a dry N_2_ atmosphere. The mixture was refluxed for 15 h until disappearance of the starting diterpene. At this point, the mixture was cooled, the solvent was evaporated, and the residue was purified by preparative TLC using hexane/EtOAc 9:1 as the eluent to obtain 24 mg (25%) of compound **8**. ^1^H-NMR (400 MHz) δ 7.37 (1H, brs, H-15), 7.26 (1H, s, H-16), 6.30 (1H, brs, H-14), 4.10 (1H, s, H-7), 3.04 (1H, s, H-5), 2.55 (2H, t, *J* = 8.5 Hz, H-12), 2.37 (1H, m, H-1), 2.25 (1H, m, H-1), 1.95 (1H, brd, *J* = 12.3 Hz, H-11), 1.82 (3H, s, H-17), 1.65 (2H, m, H-2), 1.57 (1H, m, H-11), 1.40 (1H, brd, *J* = 13.2 Hz, H-3), 1.32 (3H, s, H-18), 1.17 (1H, m, H-3), 1.02 (3H, s, H-19), 0.97 (3H, s, H-20); ^13^C-NMR (100 MHz) δ 203.9 (C-6), 148.5 (C-8), 142.9 (C-15), 138.5 (C-16), 125.5 (C-9), 124.5 (C-13), 110.6 (C-14), 62.6 (C-7), 58.2 (C-5), 46.2 (C-10), 41.8 (C-3), 36.3 (C-11), 31.9 (C-4), 31.8 (C-19), 29.4 (C-1), 24.9 (C-12), 21.9 (C-18), 21.1 (C-18), 18.6 (C-2), 16.6 (C-17); HR-EIMS *m*/*z* 334.1694 (calculated for C_20_H_27_O_2_Cl: 334.1700).

#### 3.1.8. Preparation of Solidagenone Derivative (**9**)

To a solution of 100 mg (0.32 mmol) of **1** in 5 mL of dry THF, 26 mg (2 equiv.) of NaH was added under a N_2_ atmosphere. The mixture was refluxed for 15 h, then MeOH was added and the solvent was evaporated under vacuum. The residue was purified by preparative TLC, using hexane/EtOAc 9:1 as the eluent, to yield 19 mg (20%) of compound **9**. ^1^H-NMR (400 MHz) δ 7.39 (1H, brs, H-15), 7.29 (1H, s, H-16), 6.99 (1H, brs, -OH), 6.33 (1H, brs, H-14), 2.65 (1H, m, H-1), 2.57 (3H, m, H-1, H-12), 2.05 (1H, m, H-3), 1.99 (3H, s, H-17), 1.87 (2H, m, H-2, H-11), 1.72 (1H, m, H-2), 1.56 (1H, m, H-3), 1.43 (1H, m, H-11), 1.39 (9H, brs, H-18, H-19, H-20); ^13^C-NMR (100 MHz) δ: 181.6 (C-6), 165.8 (C-7), 143.1 (C-15), 140.4 (C-5), 140.4 (C-10), 138.6 (C-16), 127.3 (C-8), 124.3 (C-13), 110.5 (C-14), 43.8 (C-9), 37.2 (CH_2_, C-11), 35.6 (C-4), 31.5 (C-1), 29.4 (C-3), 28.0 (C-19), 27.9 (C-18), 27.6 (C-20), 23.7 (C-12), 17.2 (C-2), 11.5 (C-17); HR-ESMIS *m*/*z* 337.1776 (calculated for C_20_H_26_O_3_Na: 337.1780).

#### 3.1.9. Preparation of Solidagenone Derivative (**10**)

A mixture of 100 mg (0.32 mmol) of **1** in 5 mL of DCM/MeOH 1:1 was hydrogenated in the presence of a catalytic amount of Pd/C 10% for 15 h. Then, the mixture was filtered and the solvent was evaporated under vacuum and purified by preparative TLC, using hexane/EtOAc 4:1 as the eluent, to yield 26 mg (25%) of **10**.

^1^H-NMR (400 MHz), δ (ppm): 3.92 (1H, m, H-16), 3.87 (1H, m, H-15), 3.76 (1H, dd, *J* = 15.8, 7.6 Hz, H-15), 3.36 (1H, dd, *J* = 15.3, 7.6 Hz, H-16), 2.99 (1H, s, H-5), 2.96 (1H, dd, *J* = 12.6, 8.0 Hz, H-7), 2.34 (1H, m, H-8), 2.14 (1H, m, H-13), 2.08 (1H, m, H-14), 1.95 (1H, brd, *J* = 12.6 Hz, H-7), 1.77 (1H, m, H-1), 1.52 (8H, m, H-1, H-2, H-11, H-12, H-14), 1.25 (1H, m, H-3), 1.25 (3H, s, H-18), 1.08 (1H, m, H-3), 1.03 (3H, d, *J* = 4.3 Hz, H-17), 1.00 (3H, s, H-20), 0.95 (3H, s, H-19); ^13^C-NMR (100 MHz) δ: 212.8 (C-6), 77.9 (C-9), 73.3 (C-16), 67.8 (C-15), 57.9 (C-5), 48.7 (C-10), 46.9 (C-7), 42.0 (C-3), 40.9 (C-8), 39.9 (C-13), 32.8 (C-19), 32.6 (C-11), 32.4 (C-14), 32.1 (C-4), 31.1 (C-1), 26.6 (C-12), 21.8 (C-18), 18.8 (C-20), 18.5 (C-17), 17.8 (C-2); HR-EIMS *m*/*z* 322.2514 (calculated for C_20_H_34_O_3_, 322.2508).

### 3.2. Biology

#### 3.2.1. Cell Culture Conditions

Peritoneal macrophages were obtained from Male BALB/c mice free of pathogens (8–10 weeks old) from Charles River Laboratories. All experimental protocols were reviewed and approved by the Institutional Committee for Research Ethics and were in accordance with Spanish and European guidelines legislation.

Four days prior to the assay, mice were injected intraperitoneally (i.p.) with 2.5 mL of thioglycolate broth. Elicited peritoneal macrophages were obtained as previously described [29]. Cells were seeded in RPMI 1640 containing 10% fetal bovine serum and 2% penicillin/streptomycin antibiotics.

#### 3.2.2. Determination of NO Production and Cell Viability

Peritoneal macrophages were seeded on a 96-well plate (10^5^ cells/well), stimulated with LPS (200 ng/mL), and incubated with the compounds for 24 h. Nitrite levels in supernatants were measured using the Griess reagent (1% sulfanilamide in 5% phosphoric acid and 0.1% naphtylethylenediamine dihydrochloride). Briefly, 100 µL of supernatant was mixed with an equal volume of Griess reagent and incubated at room temperature for 10 min. Absorbance was then measured at 540 nm using a microplate reader (Perkin Elmer Cetus, Foster City, CA, USA) and compared with a sodium nitrite standard curve.

To identify potential cytotoxicity of the compounds, cell viability was assessed using the MTT assay. Macrophages were seeded in 96-well plates and incubated with various concentrations of compounds for 24 h. The MTT solution (5 mg/mL) was added to the cells and the cells were incubated at 37 °C for 4 h. The reaction product, formazan, was extracted with dimethyl sulfoxide (DMSO) and the absorbance was read at 540 nm. Results are expressed as the percent reduction in cell viability compared to untreated control cultures for at least three independent experiments.

#### 3.2.3. Western Blot Analysis

Cytosolic extracts were prepared as previously described [29]. Protein content was estimated by the Bio-Rad protein assay. Protein extracts were subjected to SDS–PAGE (10–15% gels) and blotted onto polyvinylidene difluoride membranes, which were incubated with the following antibodies: total MAPKs and phosphorylated forms of p38 and ERK (Cell Signaling Technology); with anti-NOS-2, anti-IκBα, anti-IκBβ, anti-β-actin, anti-p65, and anti-PSF (Santa Cruz Biotechnology); or with anti-COX-2 (Cayman Chemical). The blots were then incubated with secondary goat anti-mouse or rabbit IgG antibodies (Cell Signaling) and developed with ECL according to the manufacturer’s instructions (GE Healthcare). β-Actin and PSF were used as loading controls.

#### 3.2.4. RNA Analysis and Quantitative PCR

Total RNA was isolated from cells with Trizol reagent (Invitrogen). Quantitative PCR (SYBRgreen) analysis was performed with an ABI7700 sequence analyzer as described [30]. Each sample was run in duplicate, and all samples were analyzed in parallel for the expression of the housekeeping gene 36B4 (acidic ribosomal phosphoprotein P0), which was used as an endogenous control for normalization of the expression level of target genes. Fold induction was determined from mean replicate values. Primer sequences are available on request.

#### 3.2.5. Protein Preparation and Docking

The X-ray coordinates of human TLR4 in complex with MD-2 and LPS were extracted from the Protein Data Bank (PDB code 4G8A). The PDB structures were prepared for docking using the Protein Preparation Workflow (Schrodinger, LLC, New York, NY, 2018) accessible from the Maestro program (Maestro, version 11.5; Schrodinger, LLC: New York, NY, 2018). The substrate and water molecules were removed beyond 5 Å, bond corrections were applied to the cocrystallized ligands, and an exhaustive sampling of the orientations of groups was performed. Finally, the receptors were optimized in Maestro 11.5 by using the OPLS3 force field before the docking study. In the final stage, the optimization and minimization of the ligand–protein complexes were carried out with the OPLS3 force field and the default value for RMSD of 0.30 Å for nonhydrogen atoms were used. The receptor grids were generated using the prepared proteins, with the docking grids centered on the center of the bound ligand for each receptor. A receptor grid was generated using a 1.00 van der Waals (vdW) radius scaling factor and 0.25 partial charge cutoff. The binding sites were enclosed in a grid box of 20 Å^3^ with default parameters and without constrains. The three-dimensional structures of the ligands to be docked were generated and prepared using LigPrep as implemented in Maestro 11.5 (LigPrep, Schrodinger, LLC: New York, NY, 2018) to generate the most probable ionization states at pH 7 ± 1 (to retain the original ionization state). These conformations were used as the initial input structures for the docking study. In this stage, a series of treatments were applied to the structures. Finally, the geometries were optimized using the OPLS3 force field. These conformations were used as the initial input structures for the docking study. The ligands were docked using the extra precision mode (XP) [31] without using any constraints and with a 0.80 van der Waals (vdW) radius scaling factor and 0.15 partial charge cutoff. The dockings were carried out with flexibility of the residues of the pocket near the ligand. The generated ligand poses were evaluated with the empirical scoring function GlideScore, a modified version of ChemScore [32]; GlideScore, implemented in Glide, was used to estimate the binding affinity and ranking of ligands [33]. The XP Pose Rank was used to select the best docked pose for each ligand. The best correlation with human TLR4 in complex with MD-2 and LPS inhibition, as well as the best values of docking score, were achieved when the 4G8A was used.

#### 3.2.6. Molecular Dynamics Simulation

The Optimized Potentials for Liquid Simulations-2005 (OPLS2005) [34] force field in the Desmond Molecular Dynamic System was used in order to study the behavior of the ligand–target complex. The resulting docking complexes were solvated with a orthorhombic box of TIP3P (Transferable Intermolecular Potential 3-Point) water [35] and counter ions were added, creating an overall neutral system simulating approximately 0.15 M NaCl. The ions were equally distributed in a water box. The final system was subjected to a MD simulation of up to 50 ns using Desmond [36]. The method selected was NPT (Noose–Hover chain thermostat at 300 K, Martyna–Tobias–Klein barostat method at 1.01325 bar with a relaxation time of 2 ps and isotropic coupling, and a 9 Å radius cutoff was used for coulombic short-range interaction), and constraints were not applied. During the simulation process, a smooth-particle Mesh–Ewald method was used to calculate long-range electrostatic interactions. For multiple time-step integration, RESPA (REversible reference System Propagator Algorithm) was applied to integrate the equation of motion with Fourier-space electrostatics computed every 6 fs, and all remaining interactions were computed every 2 fs [37]. MD simulations were carried out on these equilibrated systems for a time period of 50 ns, and frames of energy and trajectory were captured every 1.2 ps and 4.8 ps, respectively. The quality of MD simulations was assessed by the Simulation Event Analysis tool; ligand–receptor interactions were identified using the Simulation Interaction Diagram tool.

#### 3.2.7. Statistical Analysis

All numerical data were presented as means ± SD for at least three independent experiments. Statistical significance was estimated by the Student’s *t*-test for comparison between two groups. For comparison between two or more groups, one-way ANOVA followed by Bonferroni’s post-hoc comparisons were used. Differences were considered significant at * *p* < 0.05. All statistical analyses were conducted using GraphPad Prism 5.0 (GraphPad Software, CA, USA). For Western blots, a linear correlation was observed between increasing amounts of input protein and signal intensity.

## 4. Conclusions

A series of solidagenone derivatives were semisynthesized. Initial screening for anti-inflammatory potential in terms of inhibition of NO production and cytotoxicity showed that two compounds (namely **5** and **6**) were promising diterpenes. Accordingly, their mechanism of action was studied in peritoneal macrophages. Compound **5** suppressed the expression of the inflammatory enzymes NOS-2 and COX-2 and cytokine production induced by LPS in macrophages through inhibition of NF-κB and p38 signaling. In addition, this compound also inhibited inflammatory responses mediated by ligands of TLR2 and TLR3. Docking studies revealed that derivatives **5** and **6** fit very well into the large hydrophobic binding pocket of MD-2 of TLR-4. Furthermore, molecular dynamics (MD) simulations showed that **5** was additionally stabilized by hydrogen bonds between the carbonyl group present in the solidagenone scaffold and the critical polar amino acid residues Ser 120 and Arg 90, which could explain the potency of this derivative. Taken together, our results suggest that diterpene **5** might be a promising therapeutic agent in drug development for inflammatory diseases.

## Supplementary Materials

^1^H NMR (CDCl_3_, 400 MHz) and ^13^C NMR (CDCl_3_, 100 MHz) of compounds **1**–**10**.

## Abbreviations

NO	Nitric oxide
COX-2	Cyclooxygenase-2
NOS-2	Nitric oxide synthase-2
LPS	Lipopolysaccharide
TLRs	Toll-like receptors
NF-κB	Nuclear transcription factor-κB
MAPKs	Mitogen-activated protein kinases
