# Supplementary material for: Semisynthesis and Inhibitory Effects of Solidagenone Derivatives on TLR-Mediated Inflammatory Responses
Source: Molecules. 2018 Dec 4;23(12):3197. doi: 10.3390/molecules23123197 (PMC6321690; doi:10.3390/molecules23123197)

Supplementary Material

Semiynthesis and inhibitory effects on TLR-mediated-inflammatory responses of solidagenone derivatives

Irene Cuadrado[1], Ángel Amesty[2], Juan Carlos Cedrón[2], Juan Carlos Oberti[2,3], Ana Estévez-Braun[2,**], Sonsoles Hortelano[4,**] and Beatriz de las Heras[1,**]

[1]*Departamento de Farmacología. Facultad de Farmacia, Universidad Complutense de Madrid (UCM), Madrid, Spain*

[2]*Departamento de Química Orgánica, Instituto Universitario de Bio-Orgánica Antonio González, Universidad de La Laguna. Avda. Astrofísico Fco. Sánchez 2. 38206. La Laguna, Tenerife, Spain.*

[3] *Facultad de Ciencias Químicas, Universidad de Córdoba and IMBIV (UNC−CONICET), Avenida Haya de la Torre y M. Allende, Ciudad Universitaria, Córdoba, Argentina*

[4]*Unidad de Terapias Farmacológicas. Área de Genética Humana. Instituto de Investigación de Enfermedades Raras (IIER), Instituto de Salud Carlos III, Madrid, Spain.*

** Corresponding authors: Tel.: +34 913942276; fax: +34 913941726. E-mail: lasheras@farm.ucm.es, aestebra@ull.edu.es, shortelano@isciii.es.

^{1}H NMR ($CDCl_3$, 500 MHz) and ^{13}C NMR ($CDCl_3$, 125 MHz) of compounds (1-10)

^1H NMR ($CDCl_3$, 400 MHz) of compound 1

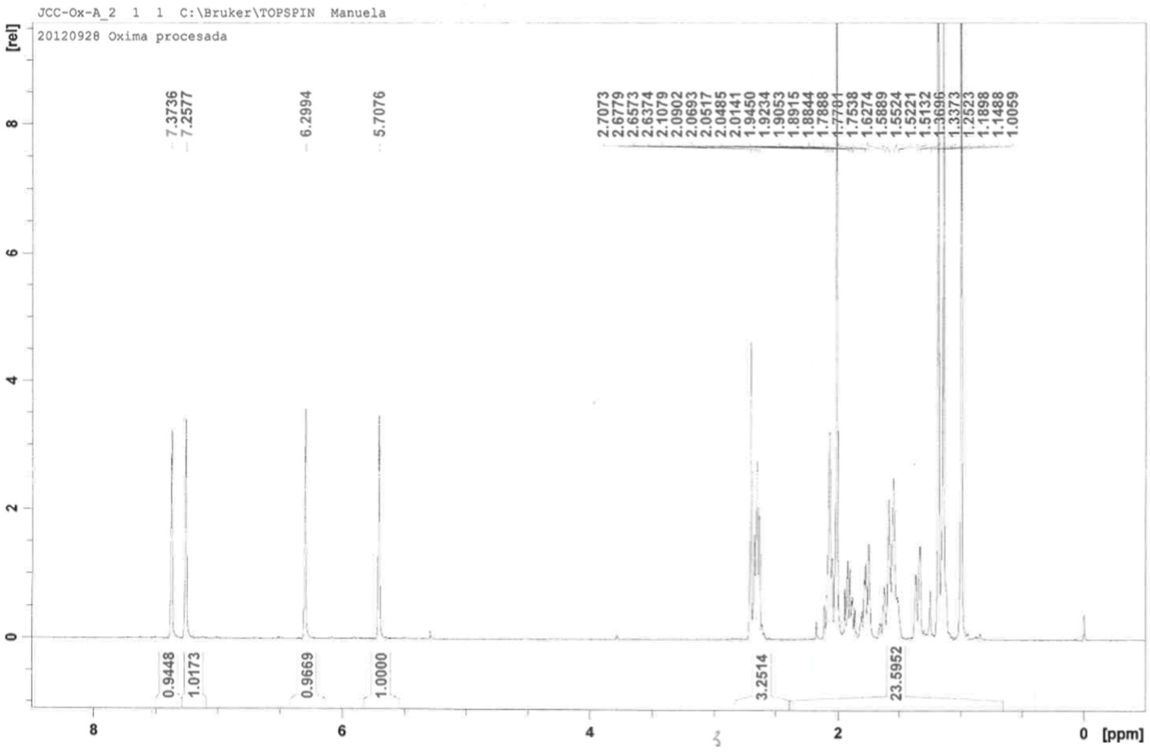

^{13}C NMR ($CDCl_3$, 100 MHz) of compound 1

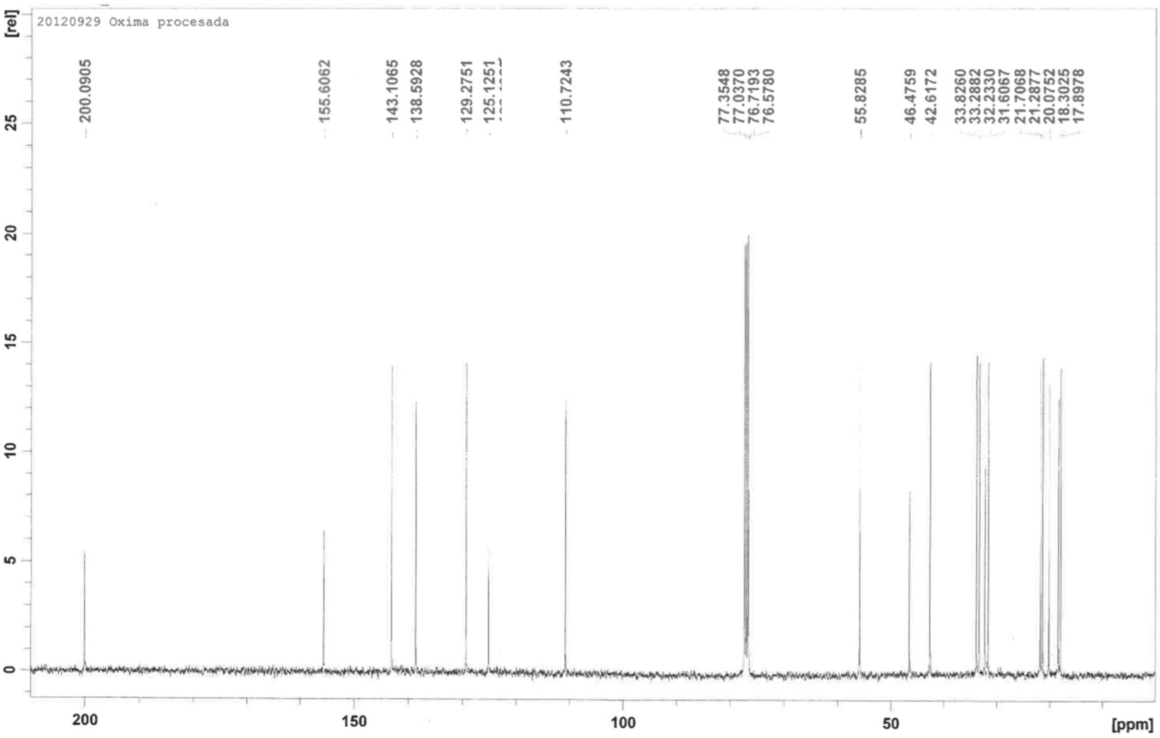

^{1}H NMR (CDCl$_3$, 400 MHz) of compound 2

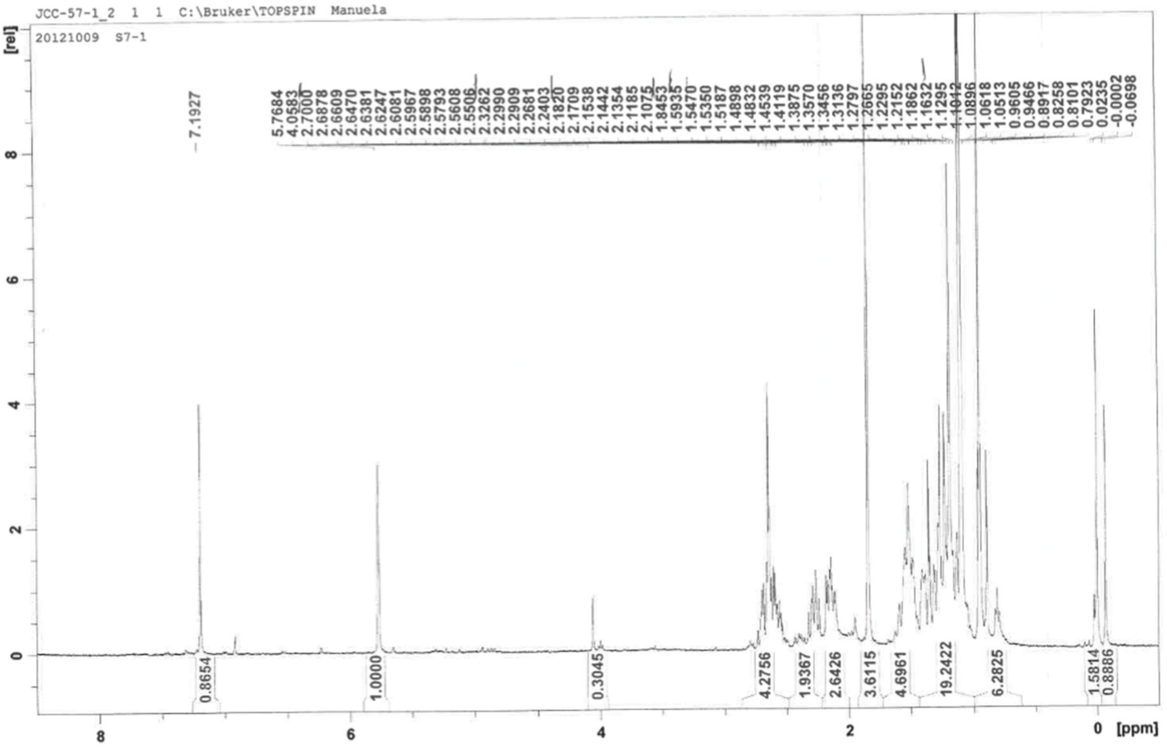

^{13}C NMR (CDCl$_3$, 100 MHz) of compound 2

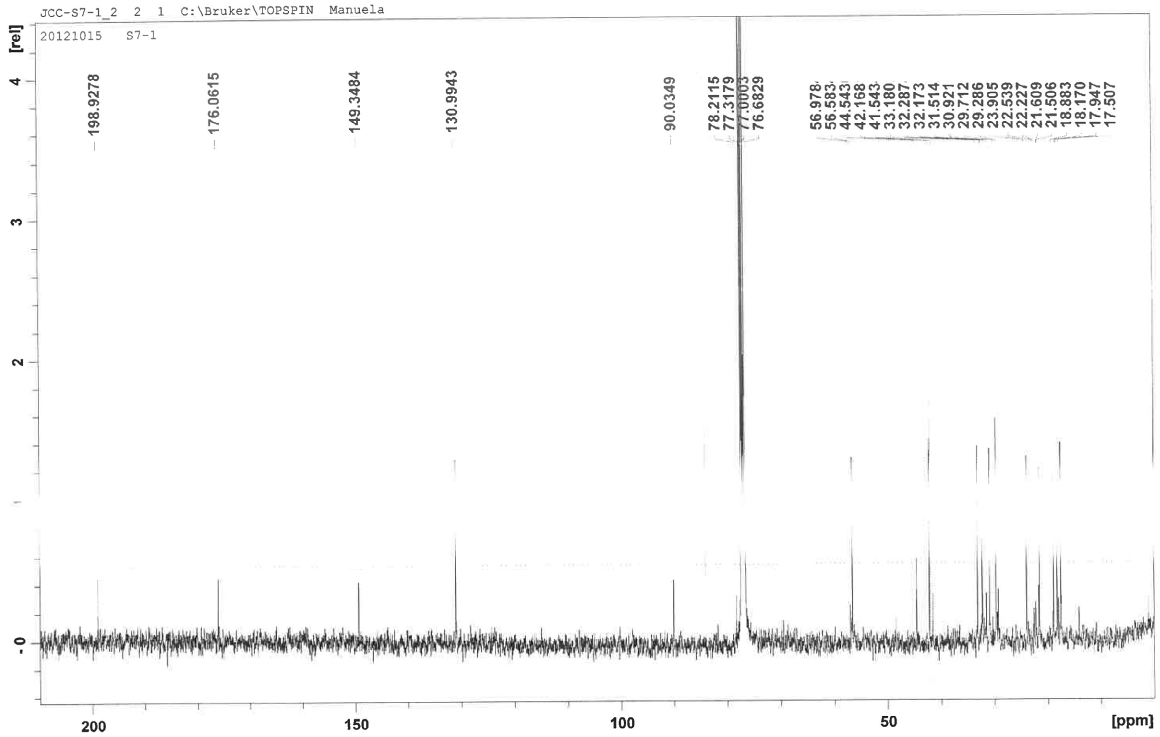

^1H NMR ($CDCl_3$, 400 MHz) of compound 3

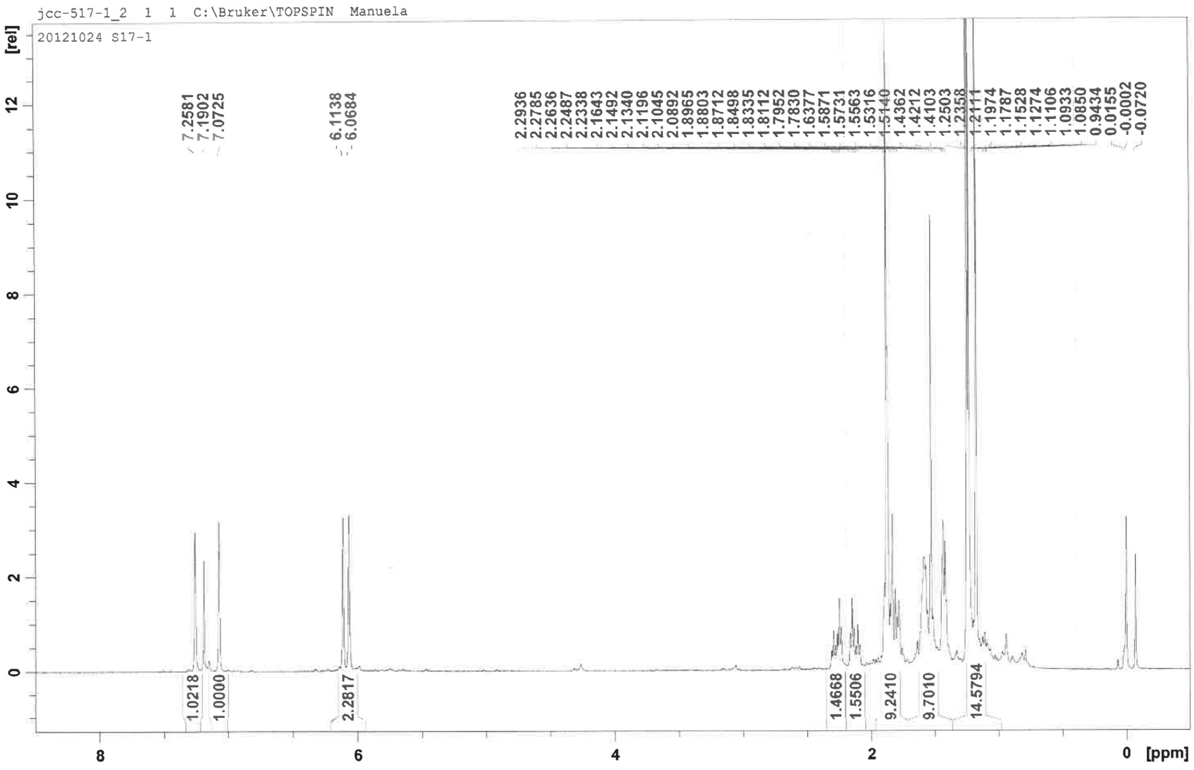

^{13}C NMR ($CDCl_3$, 100 MHz) of compound 3

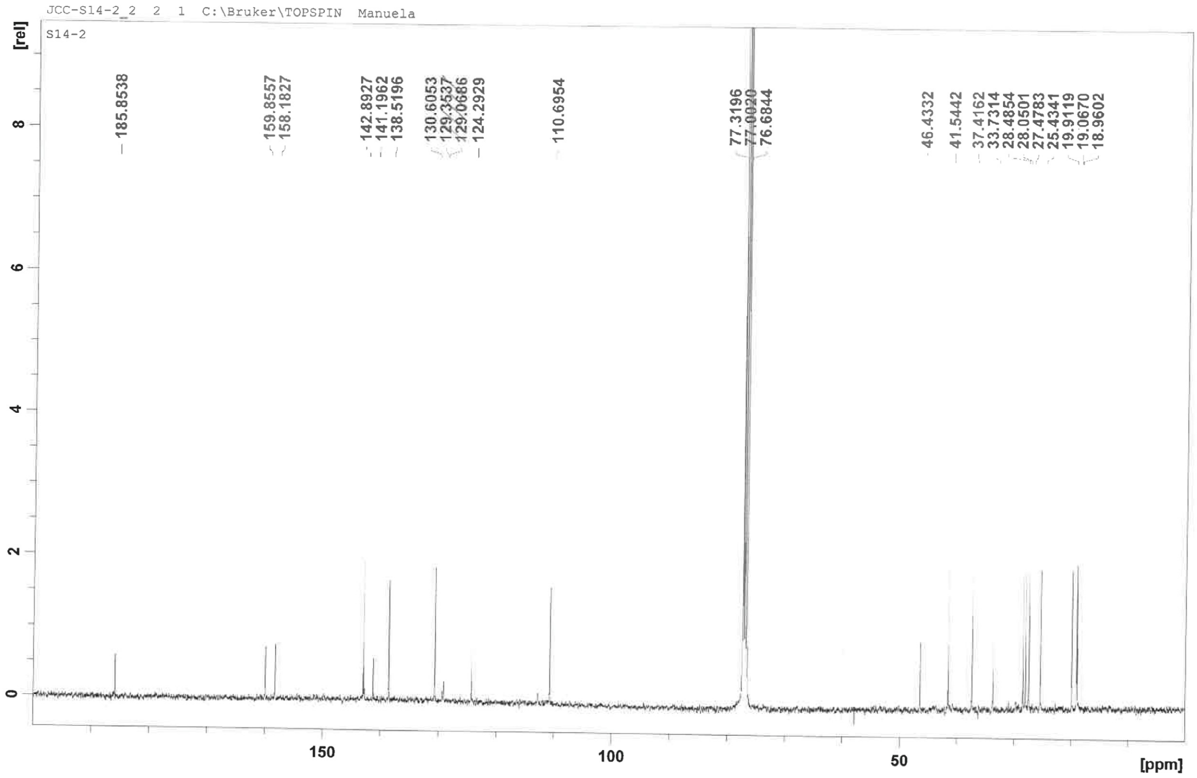

^{1}H NMR ($CDCl_3$, 400 MHz) of compound 4

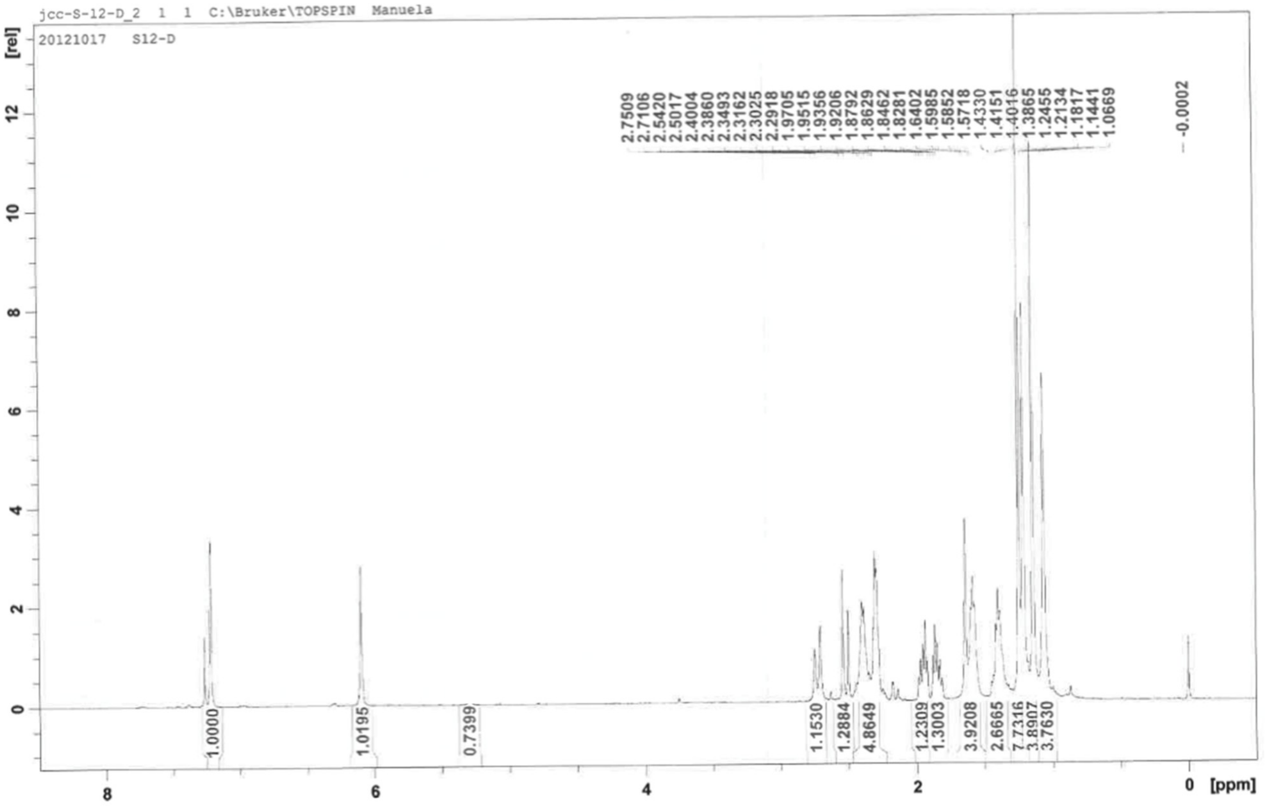

^{13}C NMR ($CDCl_3$, 100 MHz) of compound 4

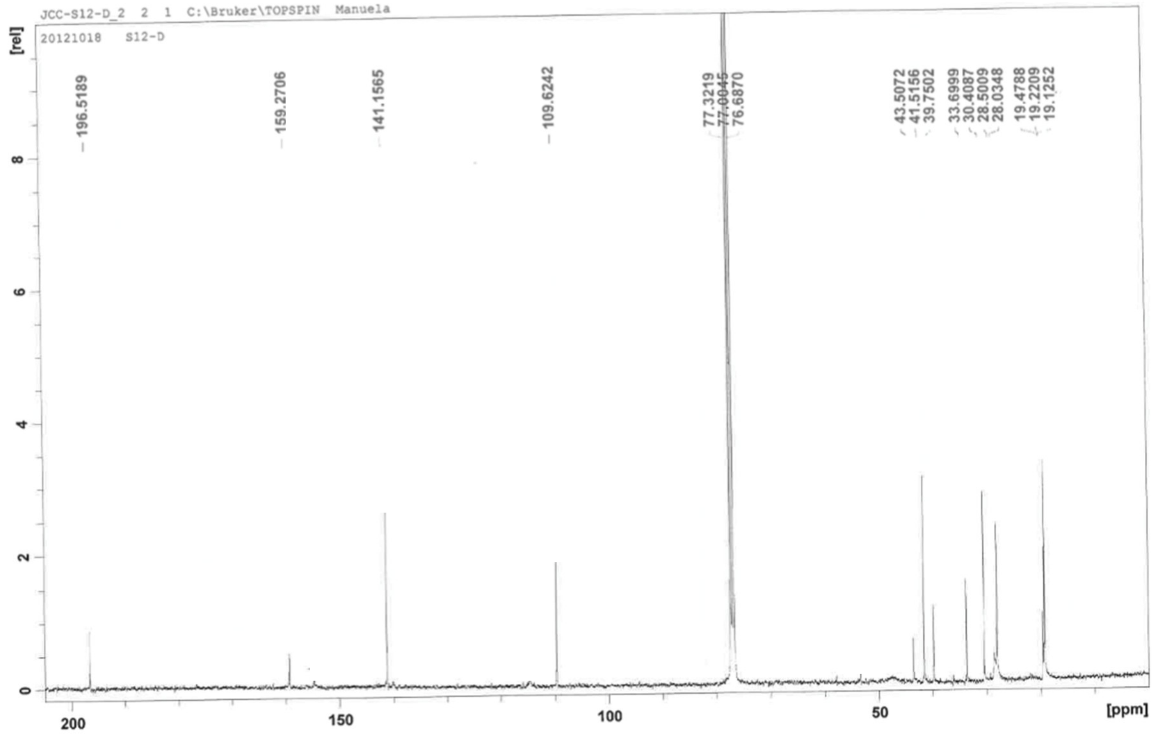

^{1}H NMR ($CDCl_3$, 400 MHz) of compound 5

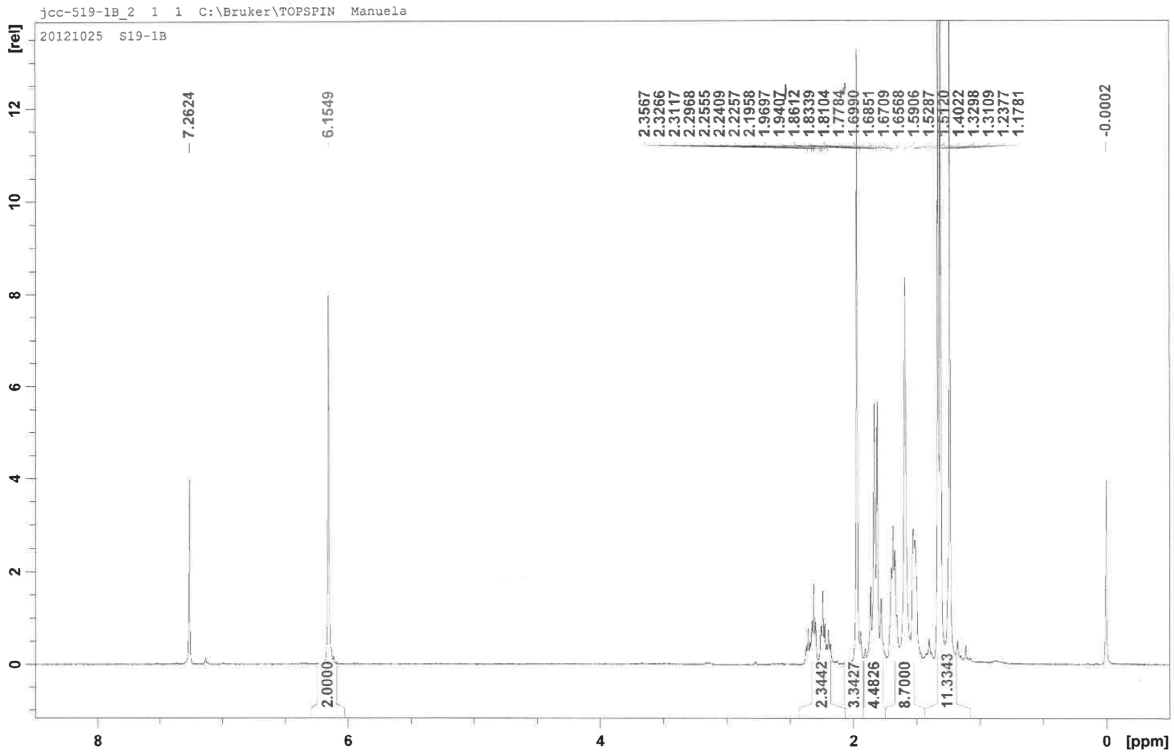

^{13}C NMR ($CDCl_3$, 100 MHz) of compound 5

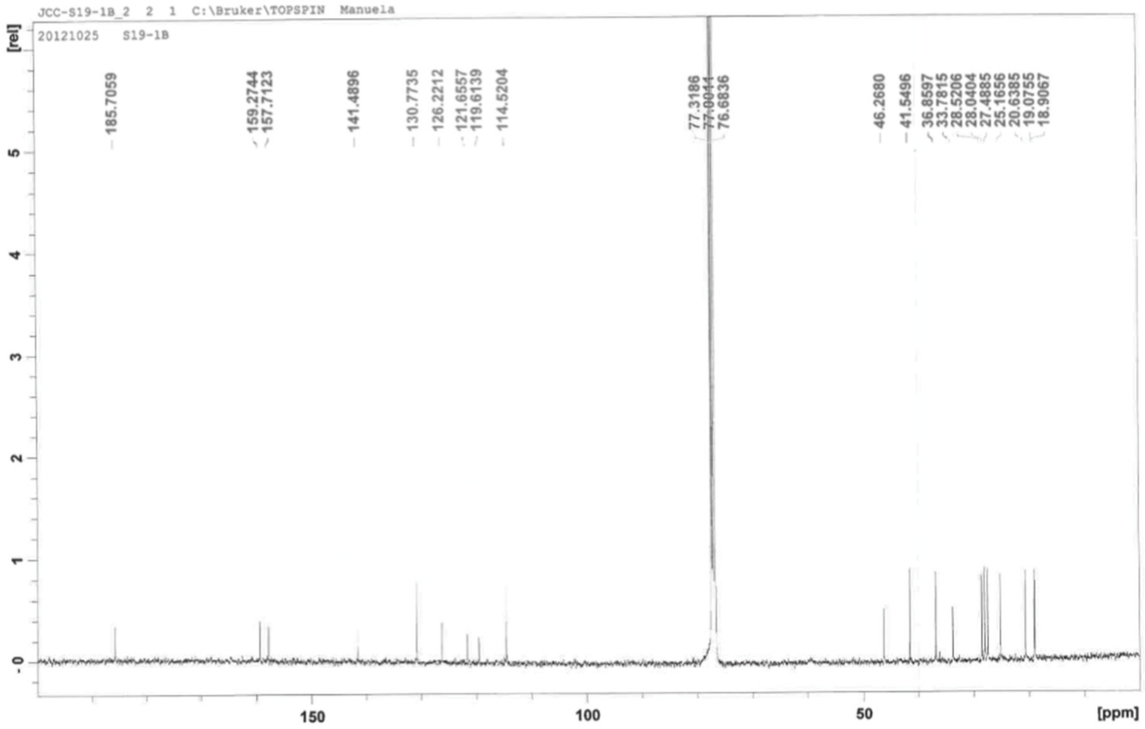

^{1}H NMR ($CDCl_3$, 400 MHz) of compound 6

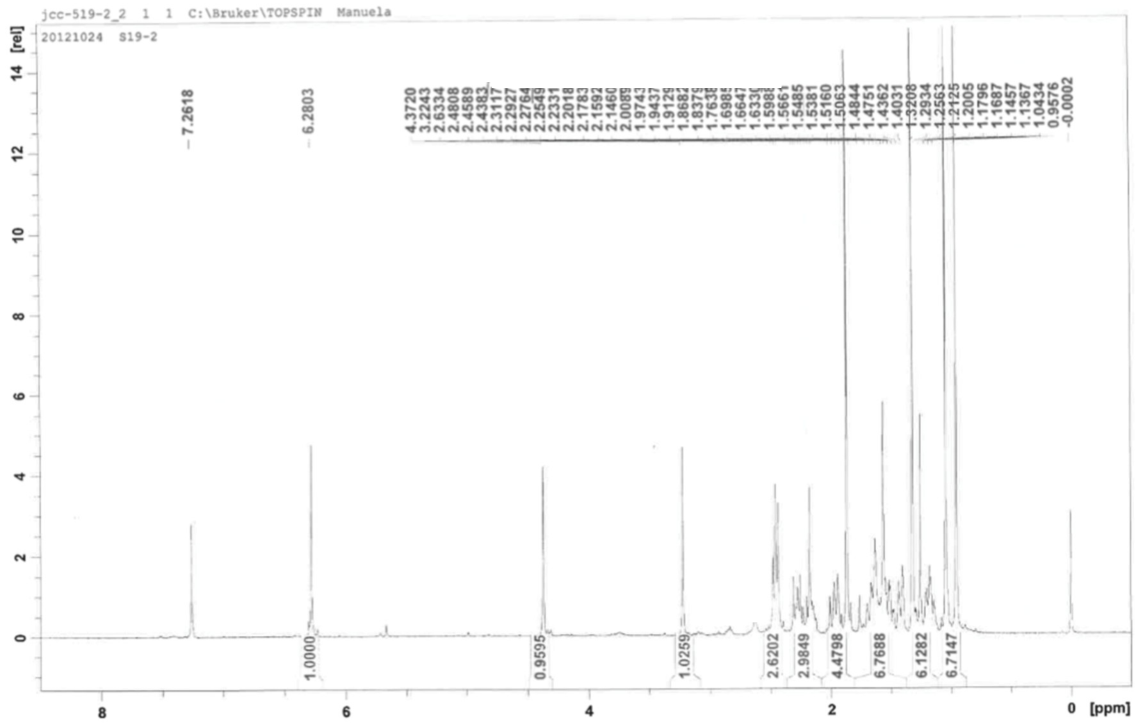

^{13}C NMR ($CDCl_3$, 100 MHz) of compound 6

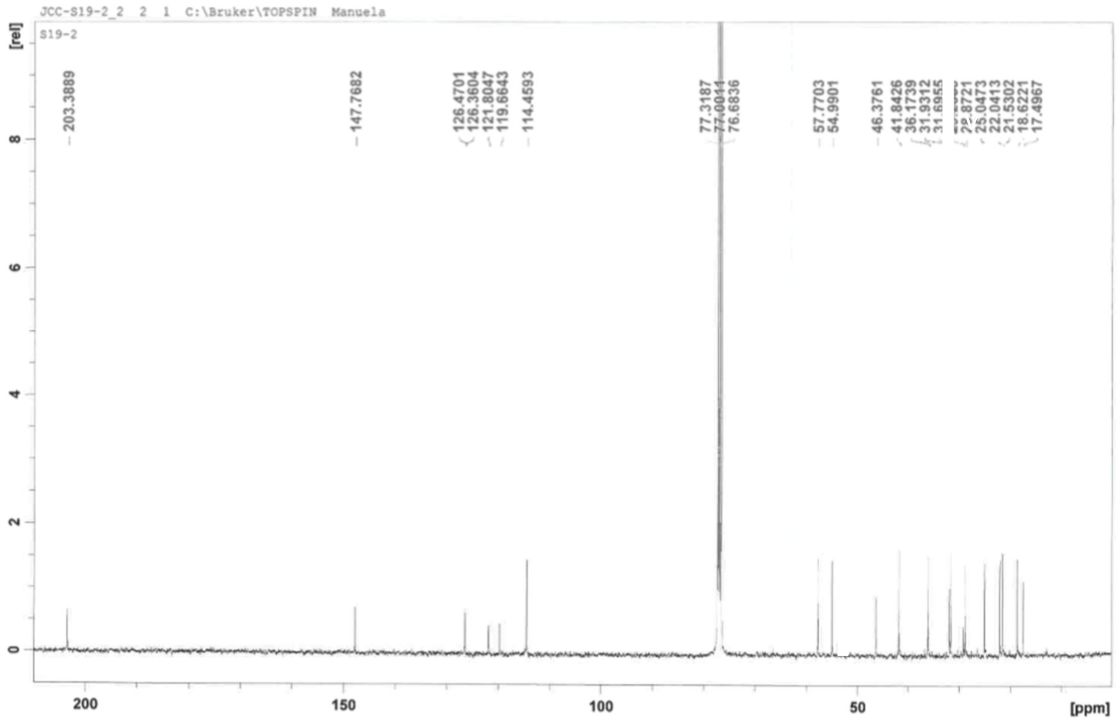

^{1}H NMR ($CDCl_3$, 400 MHz) of compound 7

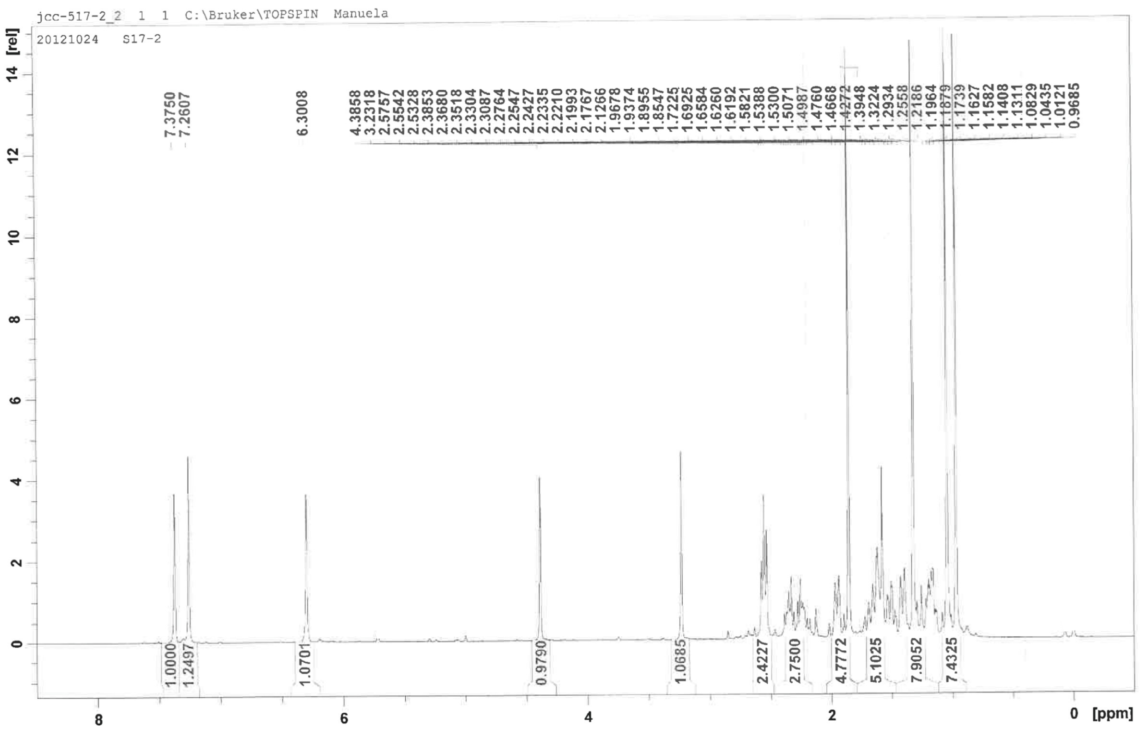

^{13}C NMR ($CDCl_3$, 100 MHz) of compound 7

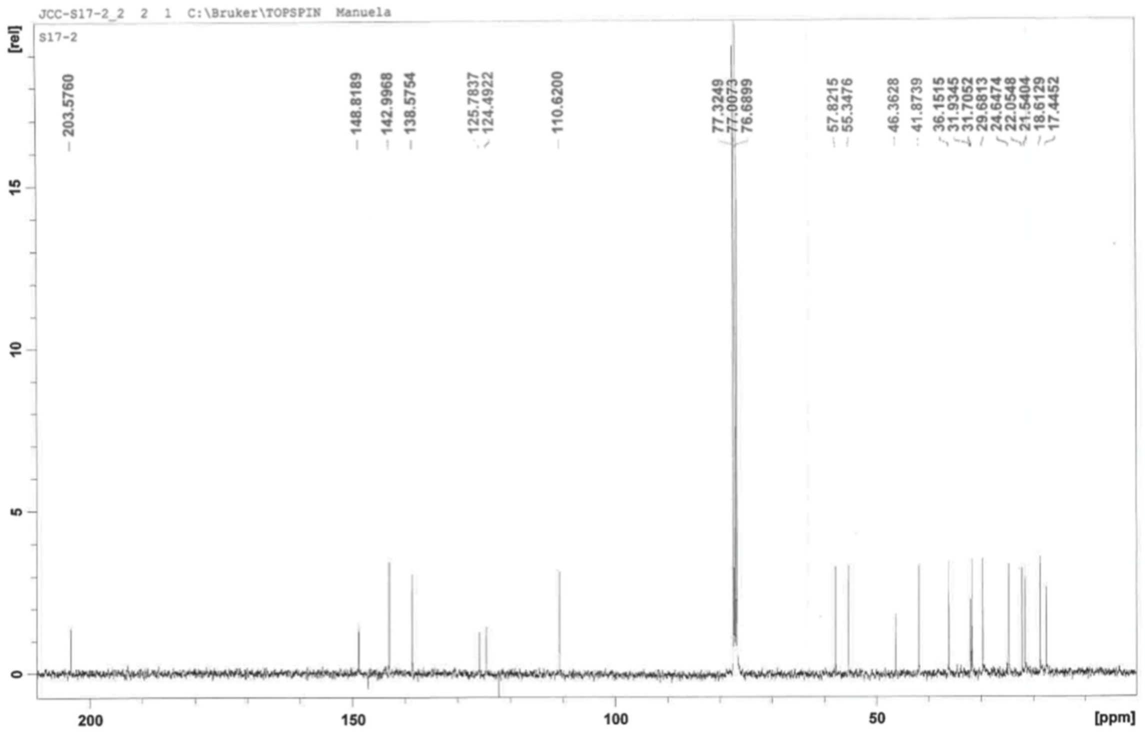

^{1}H NMR ($CDCl_3$, 400 MHz) of compound 8

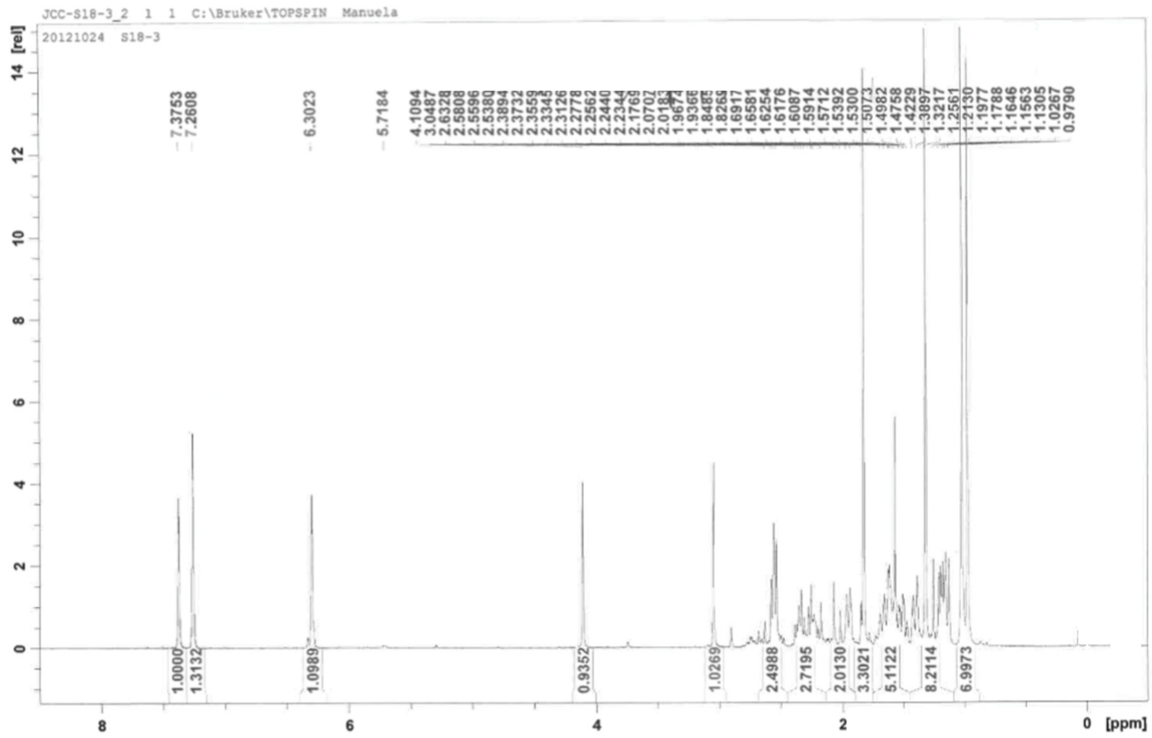

^{13}C NMR ($CDCl_3$, 100 MHz) of compound 8

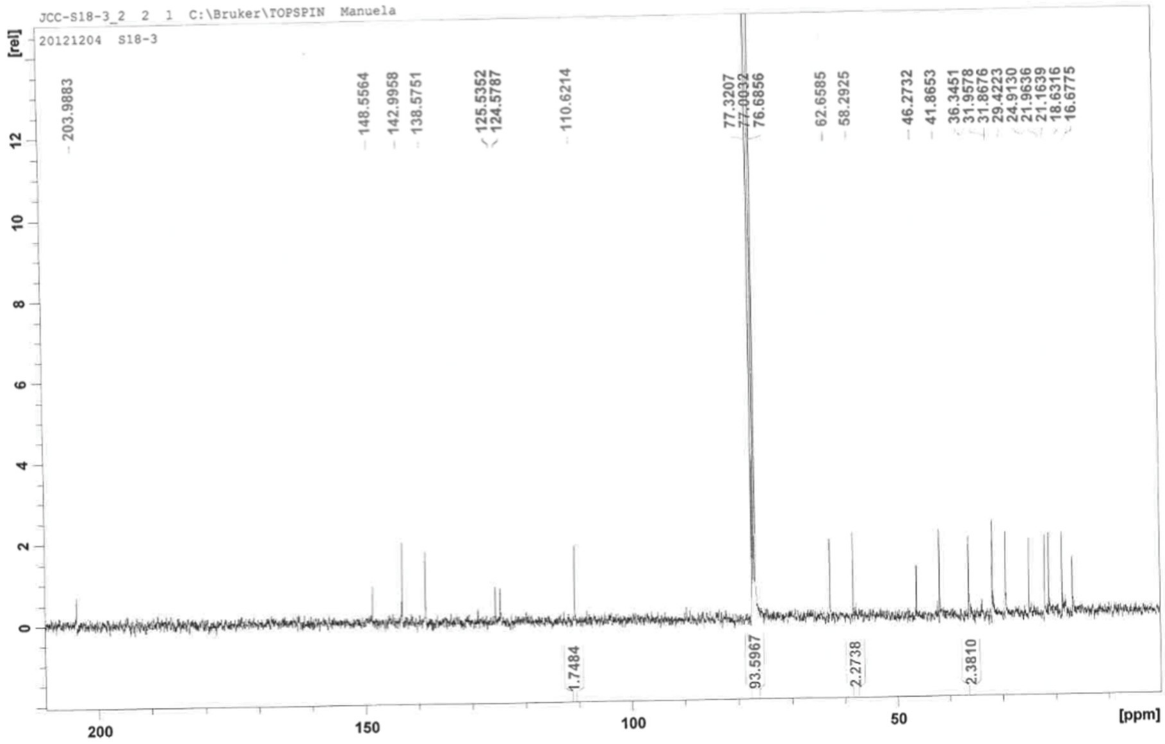

^{1}H NMR ($CDCl_3$, 400 MHz) of compound 9

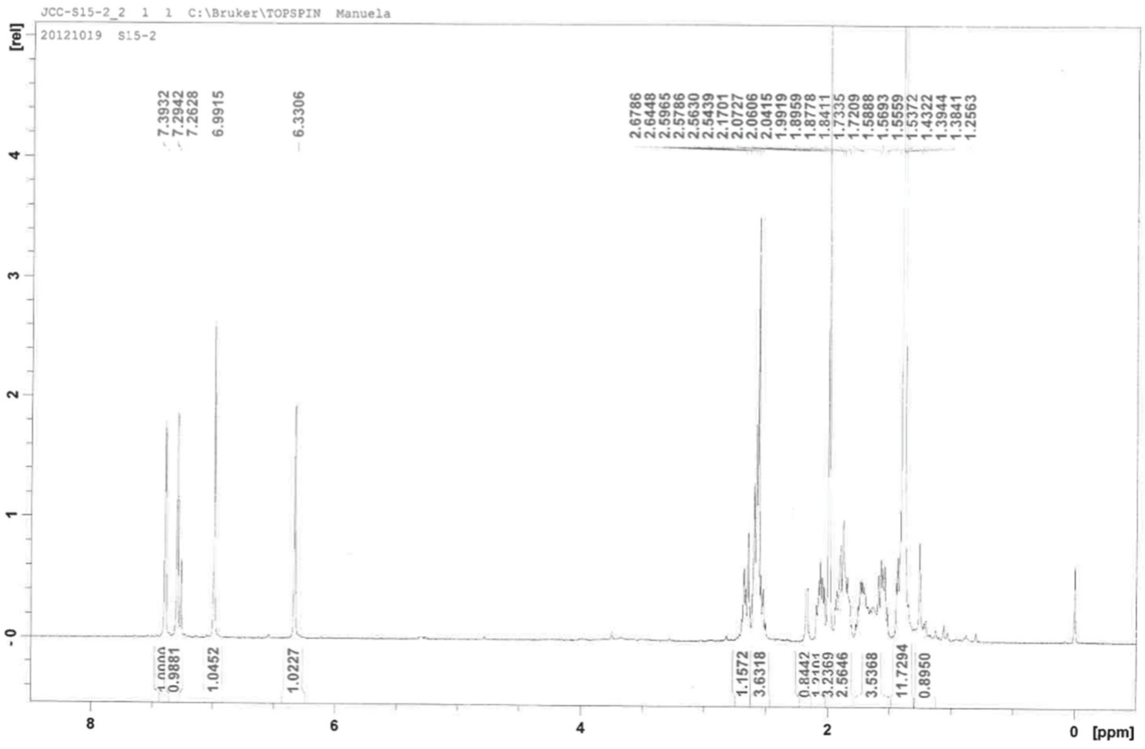

^{13}C NMR ($CDCl_3$, 100 MHz) of compound 9

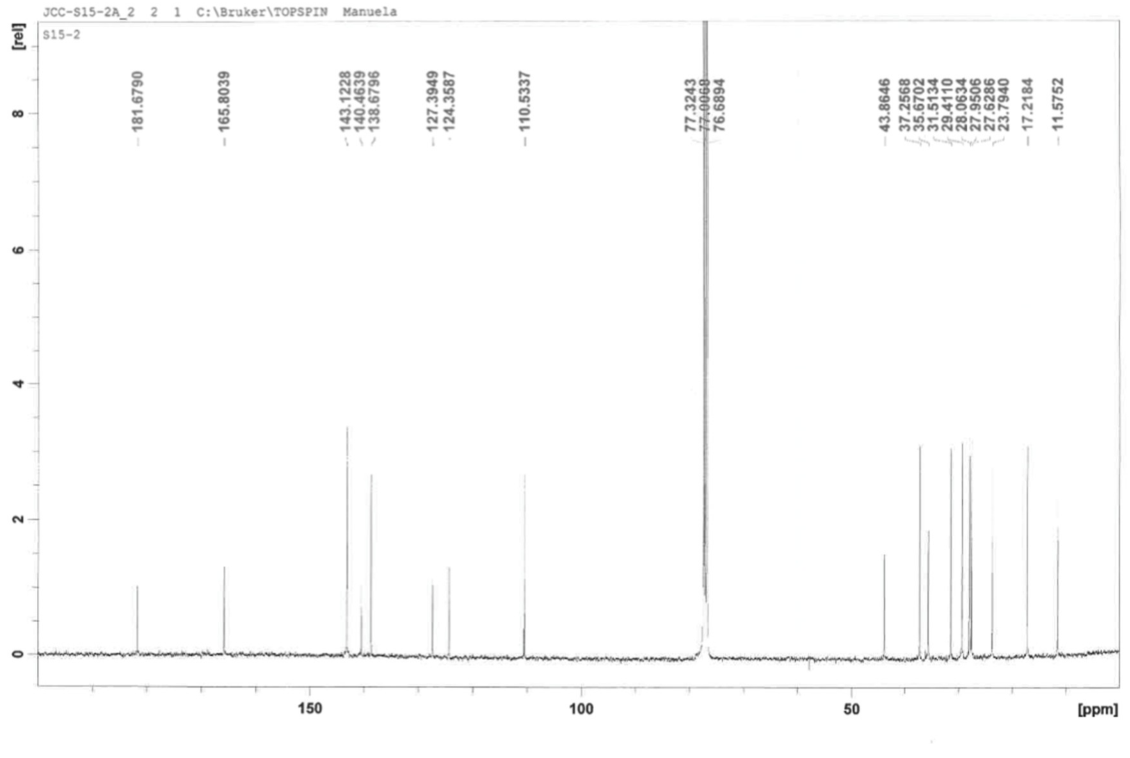

^{1}H NMR ($CDCl_3$, 400 MHz) of compound 10

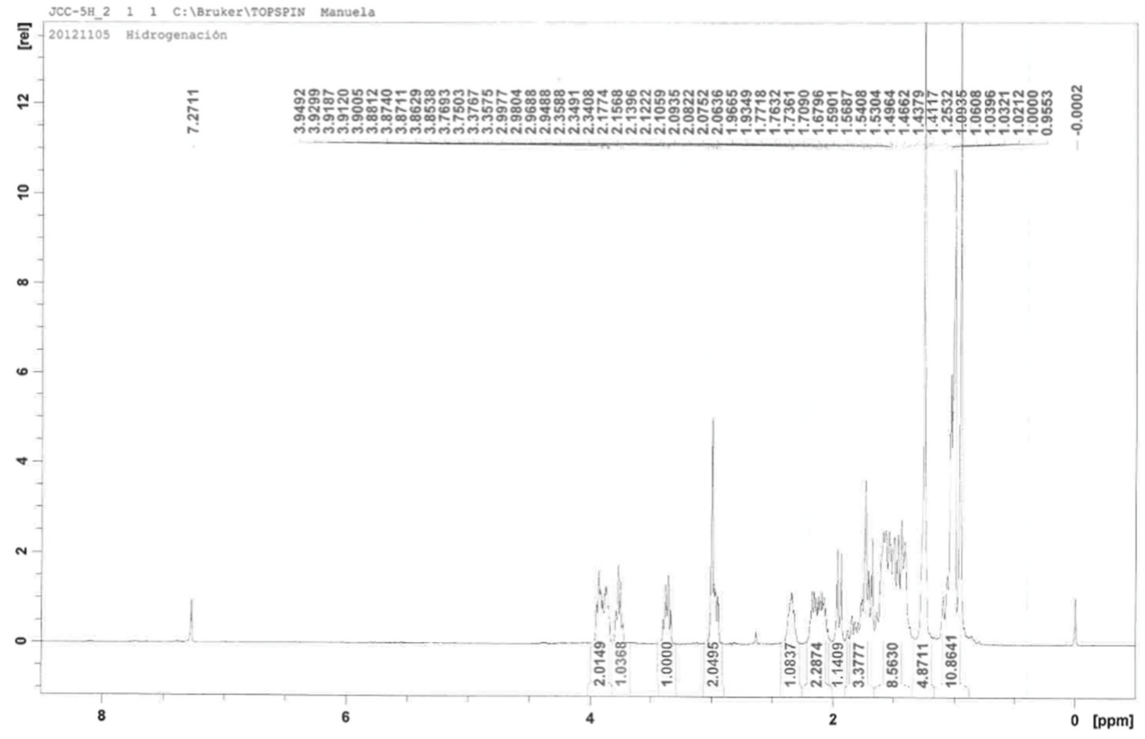

^{13}C NMR ($CDCl_3$, 100 MHz) of compound 10

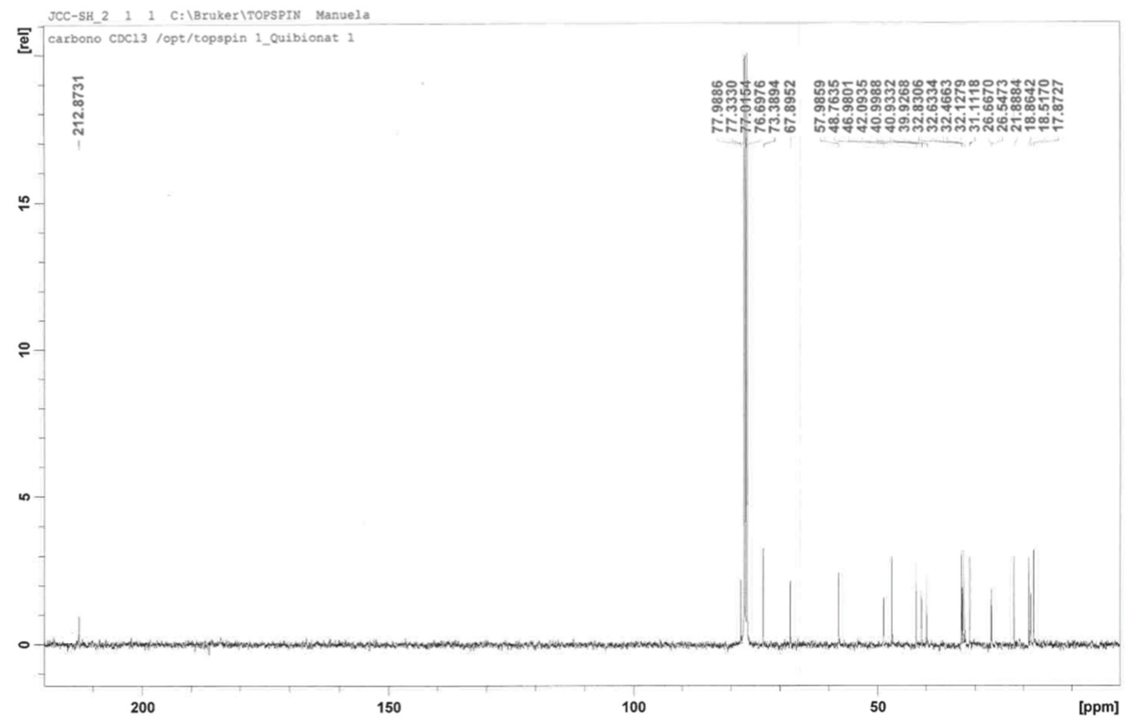

Supplement: Supplementary file 1 [file molecules-23-03197-s001.pdf]
